# Instruments measuring change in cognitive function in multiple sclerosis: A systematic review

**DOI:** 10.1002/brb3.3009

**Published:** 2023-04-16

**Authors:** Chigozie Ezegbe, Amin Zarghami, Ingrid van der Mei, Jane Alty, Cynthia Honan, Bruce Taylor

**Affiliations:** ^1^ Multiple Sclerosis Research Flagship, Menzies Institute for Medical Research University of Tasmania Hobart Tasmania Australia; ^2^ Wicking Dementia Research and Education Centre University of Tasmania Hobart Tasmania Australia; ^3^ Neurology Department Royal Hobart Hospital Hobart Tasmania Australia; ^4^ School of Psychological Sciences University of Tasmania Launceston Tasmania Australia

**Keywords:** change, cognition, impairment, measurement, multiple sclerosis

## Abstract

**Background:**

Multiple sclerosis (MS) is a chronic demyelinating/neurodegenerative disease associated with change in cognitive function (CF) over time. This systematic review aims to describe the instruments used to measure change in CF over time in people with MS (PwMS).

**Methods:**

PubMed, OVID, Web of Science, and Scopus databases were searched in English until May 2021. Articles were included if they had at least 100 participants and at least a 1‐year interval between baseline and last follow‐up measurement of CF. Results were quantitatively synthesized, presented in tables and risk of bias was assessed with the Newcastle–Ottawa Scale.

**Results:**

Fifty‐seven articles met the inclusion criteria (41,623 PwMS and 1105 controls). An intervention (drug/rehabilitation) was assessed in 22 articles. In the studies that used a test battery, Visual and verbal learning and memory were the most frequently measured domains, but when studies that used test battery or a single test are combined, Information processing speed was the most measured. The Symbol Digit Modalities Test (SDMT) was the most frequently used test as a single test and in a test battery combined. Most studied assessed “change in CF” as cognitive decline defined as 1 or more tests measured as ≥ 1.5 SD from the study control or normative mean in a test battery at baseline and follow‐up. Meta‐analysis of change in SDMT scores with seven articles indicated a nonstatistically significant –0.03 (95% CI –0.14, 0.09) decrease in mean SDMT score per year.

**Conclusion:**

This study highlights the slow rate of measured change in cognition in PwMS and emphasizes the lack of a gold standard test and consistency in measuring cognitive change at the population level. More sensitive testing utilizing multiple domains and longer follow‐up may define subgroups where CF change follows different trajectories thus allowing targeted interventions to directly support those where CF is at greatest risk of becoming a clinically meaningful issue

## Graphical abstract

This systematic review evaluated instruments used to measure change in cognitive function in large cohorts of people living with multiple sclerosis (PwMS). The results suggested that there was a lack of gold standard test and uniformity in measuring of cognitive change in PwMS.

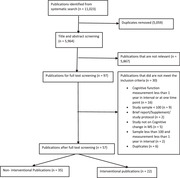

## 1 INTRODUCTION

Multiple sclerosis (MS) is an autoimmune disease that affects the central nervous system with associated demyelination, inflammation, and irreversible axonal loss seen early in the disease (Oh et al., 2018). MS can present with protean clinical features including dysfunction from the involvement of any part of the CNS with wide intra‐ and interindividual variation (Katz Sand, 2015). Females are significantly more susceptible to MS but the progression of the disease is worse in males with males getting to a specific disability level faster than females (Golden & Voskuhl, 2017).

People with MS (PwMS) can develop impairment of cognitive function in the early stages of the disease (Patti, 2009). Cognitive impairment has been observed in up to 65% of PwMS throughout the disease course (Amato et al., 2006). While cognitive impairment may be present in all types of MS, it is more common in primary and secondary progressive MS (Amato et al., 2006; Brochet & Ruet, 2019). Attention, delayed memory and executive functions are mostly affected, but language, short‐term memory and general intelligence are not typically affected (Rao, 1995). In MS, cognitive change may not be linear and may involve different domains at different points in the MS disease course. Cognitive impairment due to MS is also usually multidomain and so has multidimensional adverse effects on a person's life, which include unemployment, and problems with communication and education (Bose et al., 2022).

Most studies of cognitive functioning in MS have examined a single time point, which does not provide information about how cognition may change over time (Sumowski et al., 2018). Examining change over time is important since cognitive decline has been observed in PwMS (Eijlers et al., 2018). A decline in cognition can also be an early symptom of MS progression, secondary to potentially treatable inflammatory disease activity (Pitteri et al., 2017; Shanmugarajah et al., 2017). Therefore, there may be need for a comprehensive assessment by highly trained clinicians (neuropsychologists), indicated by cognitive impairment on screening, to monitor change in cognitive function. But this is often not feasible given the lack of specialist clinicians availability, and because it is expensive, complex and time‐consuming to undertake (Longley & Honan, 2022). However, early detection is essential given the potential for a “brain healthy lifestyle” to protect against further decline and the availability of rehabilitation to improve cognitive functioning or assist with the development of cognitive compensation strategies (Longley & Honan, 2022; Meca‐Lallana et al., 2021).

Several clinical tools have been developed to assess cognitive function and/or screen for possible cognitive impairments specifically in MS. These are mostly pen and paper validated clinical tools and include cognitive test batteries such as the Minimal Assessment of Cognitive Function in MS (MACFIMS), Rao's Brief Repeatable Neuropsychology Battery (BRNB), Medical Outcomes Scale‐Cognitive Functioning (MS‐COG), Brief International Cognitive Assessment for MS (BICAMS), Multiple Sclerosis Functional Composite (MSFC) (Sumowski et al., 2018), and MSReactor (Merlo et al., 2019). Screening tools are easy to administer, easy to interpret with the use of cut‐off scores, reflect the cognitive functioning of PwMS at a general level, and can be administered repeatedly to monitor change in CF though cognitive impairment in people with above average baseline levels of intellectual functioning could be missed (Longley & Honan, 2022). Practice effect (more common between first and second assessments) can be an issue and can be minimized through dual baseline assessment before further regular assessments (Duff et al., 2001). The heterogeneity of these batteries which includes having different tests measuring similar domain makes the comparison of change in cognitive function problematic.

Although each cognitive test is usually regarded as measuring a particular cognitive domain, they also tap into abilities in other functional domains. For example, while the Symbol Digit Modalities Test (SDMT) is primarily considered a test of information processing speed, it also requires abilities in visual scanning and incidental memory (Sumowski et al., 2018). The SDMT, written or oral form, nonetheless has been observed to be a reliable and valid test for the measurement of information processing speed in PwMS (Benedict et al., 2017) and slowed processing speed is considered a hallmark characteristic of MS due to the loss of myelin on neurons that facilitate neural communication (Sivakolundu et al., 2020). Experts convened by The US National MS Society recommended that the SDMT, or similarly validated test, be used for an annual assessment of cognitive function in PwMS as it is sensitive to cognitive function changes and the best rapid clinical assessment tool for cognitive function (Kalb et al., 2018). Therefore, the SDMT may be referred to as the current gold standard to assess cognitive function in MS.

This systematic review aims to evaluate how changes in cognitive function occur in PwMS, over a minimum 1‐year observation period, and how this change was measured and quantified, in studies that included samples of at least 100 participants (PwMS and healthy controls inclusive). The rationale in choosing these studies is to understand how change in cognitive function in PwMS occurs, and how it is measured, at a population or epidemiological level. The review will include details of the assessment tools used, the cognitive domains these tools measure, and the extent of change over time reported in studies using these tools. A meta‐analysis examining change in SDMT scores over time is also presented.

## 2 METHODS

### 2.1 Search strategy

PubMed, OVID, Web of Science, and Scopus were searched using the keywords “chang*” OR “trend*” OR “increase” OR “decrease” OR “reduc*” OR “time” OR “follow‐up” OR “longitudinal” OR “cohort” and “cogniti* function*” OR “cogniti* dysfunction*” OR “cogniti* impairment” OR “cogniti*” OR “memory” OR “cogniti* decline” OR “cogniti* deficit” OR “processing speed” OR “attention” OR “executive function*” and “Multiple Sclerosis.” The search was undertaken on the May 31, 2021 and included all studies on change in cognitive function in MS without a date or MS patient group (relapsing‐remitting MS (RRMS), primary progressive MS (PPMS), secondary progressive MS (SPMS)) restriction. Only articles published in English and on humans were included. This systematic review was registered in PROSPERO: Registration CRD42021255389.

### 2.2 Study selection

Title and abstract screening was completed by one rater (CE) in Covidence using the following exclusion criteria: the study did not measure cognitive function in MS, measured cognitive function at only one time point, measured cognitive function at less than a 1‐year interval between baseline and follow‐up, had less than 100 participants (study control inclusive), or were duplicates, case reports, brief reports, letters to the editor, reviews, or a study protocol. The full‐text screening was completed by two researchers (CE and AZ) using the stated criteria working independently. Conflicts were resolved by consensus or by a third researcher (BT) when the first and second researchers could not reach a consensus.

### 2.3 Data extraction

Data were extracted by one reviewer (CE) with the following information: author, year of publication, country, cognitive domain measured, corresponding cognitive test or battery, maximum interval between baseline and follow‐up measurement, study sample size, age range of participants, patient group (RRMS, PPMS, and SPMS), MS duration (mean, median, range), baseline EDSS, how a change in cognitive function was measured, study findings related to cognitive function, and the intervention/s used (if applicable). Although the information on many cognitive domains and tests was extracted, only studies with SDMT mean score and standard deviation at baseline and follow‐up and sample size at baseline and follow‐up were used for the meta‐analysis. Fifteen authors were contacted through email for some missing information for the meta‐analysis but five responded. Only papers with complete data were used for the meta‐analysis. SDMT was chosen because it is sensitive to cognitive function changes and has been recommended as the best clinical practice tool for the assessment of cognitive function in PwMS (Kalb et al., 2018).

The quality and risk of bias assessment of the included studies was assessed by 2 reviewers (CE and AZ) independently using the Newcastle–Ottawa Scale (NOS). Stars were assigned to the included publications based on three criteria: selection of cases/controls or cohorts, comparability of cases/controls or cohorts, and outcome assessment with a maximum of four, two and three stars respectively. For each publication, total stars ranging from 0 to 3, 4 to 6, and 7 to 9 were considered as low (high risk of bias), moderate (moderate risk of bias), and high quality (low risk of bias), respectively.

### 2.4 Statistical analysis

A meta‐analysis was completed using STATA version 17 to examine change over time in SDMT scores. The SDMT has been recommended as one of the most reliable and valid tools for measuring cognitive function in MS. Cohen's d of SDMT between baseline and follow‐up was calculated for all studies. A weighted average was applied depending on the sample size and the random‐effects model used due to study heterogeneity. The analysis was reported with a forest plot (with 95% confidence intervals). A negative value in the effect size indicated a decline in cognitive function whereas a positive value indicated improvement. A *p*‐value of < .05 was considered statistically significant. The heterogeneity of the studies used for meta‐analysis was assessed with *I*2 with values of 25%, 50%, and 75% considered as low, moderate, and high, respectively (Higgins et al., 2003). Publication bias was assessed with Egger's test.

## 3 RESULTS

**FIGURE 1:**
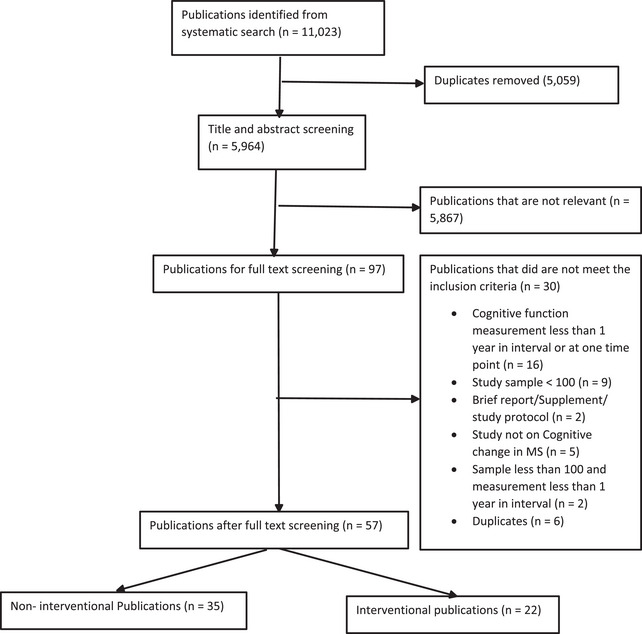
Flowchart of the literature search and study selection process.

The primary search yielded 11,023 publications. After duplicates were removed, 5964 remained. Title and abstract screening yielded 97 papers. After full‐text screening, 57 papers were included (Figure 1) of which 35 were observational studies (Table 1) and 22 were interventional studies (Figure 1 and Table 2). All the studies were published between 1995 and 2021. North America contributed 22 papers, Europe 32, Oceania 2, and Asia 1. The sample size of PwMS and controls in the articles ranged from 100 (Raimo et al., 2020) to 11,222 (Crielaard et al., 2019) and included a total of 41,623 PwMS and 1105 controls. The interval between cognitive function measurement at baseline and follow‐up ranged from 1 to 30 (Crielaard et al., 2019) years. Five (Demakis & Buchanan, 2010; Demakis et al., 2009; Hughes et al., 2018; Lincoln et al., 2020; McKay et al., 2019) out of the 57 included studies did not specify the Expanded Disability Status Scale (EDSS) of their study participants. Among studies that specified the EDSS mean or median, all had a mean or median of less than 6.5 except for one study that reported participants’ EDSS in the range of 6.0 to 9.5 (Ytterberg et al., 2008). The mean age ranged from 12.5 (Amato et al., 2010) to 57.5 years (Demakis & Buchanan, 2010) for all participants—PwMS and controls. All MS groups in the included studies reported a mean disease duration of 6 months (Johnen et al., 2019) to 21 years at baseline (Chan et al., 2017). After assessment of the risk of bias for all the 57 included publications, five were of high quality (7 to 9 stars/low risk of bias) while the rest were of moderate quality (4 to 6 stars/moderate risk of bias) (Supplementary TablesS1 and S2).

**TABLE 1:** Characteristics of studies measuring change in cognitive function in people living with multiple sclerosis without intervention.

Author and year	Location	Cognitive domain measured	Maximum gap between measurement of cognition (years)	Study population	Age (at baseline) Mean (SD) years	Patient group (RRMS, PPMS, SPMS) at baseline	MS duration (MEAN) at baseline: Mean (SD) years	Definition of cognitive impairment (or improvement)/change in cognitive function	EDSS score (mean) at baseline: Mean (SD)	Findings about cognitive function
Amato et al. (2010)	Italy	Verbal learning and delayed recall, visuospatial learning, complex attention, planning, expressive language	2+	106: 56 PwMS and 50 HC	PwMS: (Age 10–15: 12.5 (1.3), Age ≥ 15: 17.9 (1.8)) HC: (Age 10–15: 11.2 (1.8), Age ≥ 15: 18.6 (2.4))	56 RRMS	5.5 (3.7)	< –2 SD in three or more cognitive tests (study controls as norms)/ comparison of the proportion of cognitively impaired/improved participants between baseline and follow‐up	1.7 (1.0)	75% of PwMS had a decline in cognitive function compared to the study control. These were outstanding in verbal memory, complex attention, receptive language, and verbal fluency
Amato & Ponziani (1998)	Italy	Memory, learning and recall, abstract reasoning and attention, acquisition, and recall	4.53	120: 50 PwMS (32 women and 18 men) and 70 HC	NA	44 Relapsing‐remitting, 6 chronic progressive	1.58 (1.62)	< –2 SD on ≥5 cognitive tests (study controls as norms) / comparison of the proportion of cognitively impaired/improved participants between baseline and follow‐up	1.98 (1.48)	PwMS scored lower (statistically significant) than the healthy controls on all tests, except the information‐memory‐concentration (IMC). Cognitive decline predicted poor work outcomes and social activities
Amato et al. (1995)	Italy	Executive functions	4+	120: 50 PwMS (18 males and 32 females) and 70 HC	PwMS: 29.9 (8.48) HC: 29.3 (1.15)	44 RRMS and 6 PMS	1.58 (1.62)	← 2 SD on a cognitive test (study controls as norms))/ comparison of the proportion of cognitively impaired/improved participants between baseline and follow‐up	2.55 (2.41)	PwMS scored lower than HC at baseline and follow‐up. There was a statistically significantly higher number of failed subtests at baseline and follow‐up in PwMS compared to HCs.
Amato et al. (2001)	Italy	Memory, learning (recall), abstract reasoning and attention, acquisition, and recall	5	120: 50 PwMS (32 women and 18 men) and 70 HC (44 women and 26 males)	PwMS: 29.90 (8.48) HC: 29.30 (5.17)	Relapsing‐remitting (44), Chronic progressive (6)	11.34 (2.31)	< –2 SD on ≥ 3 cognitive tests (study controls as norms)/ comparison of the proportion of cognitively impaired/improved participants between baseline and follow‐up	1.98 (1.48)	People living with MS performed worse than the controls. Physical disability, disease progression, and older age predicted cognitive decline
Beckerman et al. (2013)	The Netherlands	Comprehension, expression, social interaction, problem‐solving and memory	10	156 PwMS (55 males and 101 females)	Median (IQR): 37.1 (29.6, 45.2)	Relapse onset (128) and nonrelapse onset (28)	10	Change in score over time (between relapse or nonrelapse type MS)	Median (IQR): 2.5 (2.0, 3.0)	There was a 10% change or 3‐point change in 10 years in FIM cognitive function scale in all PwMS
Bosma et al. (2010)	Netherlands, Spain and United Kingdom	NA	2	161 PwMS (sex ratio not defined)	Not defined	All primary progressive (PP) MS	Not defined	15% and 20% mean change in score between baseline and follow‐up.	Range: 2.0, 6.5	Different cut‐off points for cognitive decline may need to be applied for different forms of MS. PASAT is suggested as not a good scale to measure disease progression.
Borghi et al. (2016)	Italy	Verbal memory (learning and delayed recall, learning and delayed recall, attention, speed of information processing, working memory, sustained attention, and verbal fluency)	2	625: 322 PwMS (97 males and 225 females) and 303 HC (93 males and 210 females)	41.98 (11.37)	89% of the PwMS participants had RRMS while 11% had progressive MS	9.16 (7.13)	< –1.5 SD on ≥ 2 cognitive tests (study controls as norms)/ comparison of the proportion of cognitively impaired/improved participants between baseline and follow‐up	2.37 (1.90)	The proportion of PwMS that had cognitive impairment increased at 1 year but came back to the baseline level at 2 years
Bsteh et al. (2019)	Austria	NA	3	151 RRMS (32 males and 119 females)	35.1 (9.4)	All RRMS	5.8 (2.7)	≥4‐point loss or > 10% reduction in SDMT score between baseline and follow‐up	Median (range): 1.5 (0, 6.5)	Peripapillary retinal nerve fiber layer thickness ≤88 μm was associated with a 2.7‐fold increased risk of cognitive function decline in the following 3 years
Chruzander et al. (2014)	Sweden	Attention	10	118 PwMS: 33 males and 85 females	49 (11)	55 RRMS, 63 Progressive	18 (11)	← 1.5 SD from the mean score of a test)/ comparison of the proportion of cognitively impaired/improved participants between baseline and follow‐up	EDSS: 0−3.5 (41 PwMS), 4.0−5.5 (24 PwMS), 6.0−9.5 (51 PwMS)	There was a decline in cognitive function between baseline and follow‐up (not statistically significant). Cognitive impairment predicted worse health‐related quality of life
Crielaard et al. (2019)	Sweden	Cognitive processing efficiency and speed	30	11 222 PwMS	Benign: 28.3 (8.5) Nonbenign: 32.9 (10.4)	All RRMS	Benign: 14.3 (9.7) and Nonbenign: 6.8 (9.5)	SDMT trajectory compared	Benign: (Median (IQR)) –2 (1, 2.5) and Nonbenign: (Median (IQR)) –2 (1, 4.0)	There was an associated 0.3 (95% CI: −0.39 to −0.18, *p* < .001) decline in SDMT raw score per year in nonbenign patients
de Groot et al. (2009)	The Netherlands	Verbal learning and memory, visuospatial learning and delayed recall, sustained attention, concentration, information processing speed and verbal fluency	3	146 PwMS: 53 males and 93 females	37.4 (9.7)	Not defined	< 6 months	< –1 SD on ≥1 cognitive tests (population controls as norms) / comparison of the proportion of cognitively impaired/improved participants between baseline and follow‐up	Median (IQR): 2.5 (2.0, 3.0)	Age and gender and T2‐ weighted supratentorial lesion load are predictors of cognitive impairment
De Meijer et al. (2018)	Australia	Psychomotor function, information processing speed, attention, visual learning, spatial problem‐solving, executive function and working memory, attention, and calculation ability.	1	106: 66 PwMS (14 males and 52 females) and 40 HC (12 males and 28 females)	PwMS: 47.5 (12.4) and HC: 35.2 (14.7)	49 for relapsing‐remitting multiple sclerosis, 17 for secondary progressive multiple sclerosis, and 40 for healthy controls	14.0 (10.8)	Not defined	Median (IQR): 2.5 (1.25, 4.0)	Cognitive scores of PwMS were significantly worse compared to HCs. CogState Brief Battery was more sensitive and acceptable and with less anxiety (based on scoring) compared to PASAT.
Demakis & Buchanan (2010)	United States	Short‐and long‐term memory, orientation, and communication	3	924 PwMS: 72% female and 28% male	57.5 (13.5)	MS (MS without psychiatric or neurological comorbidities), MS‐Neuro (MS with neurological comorbidities), MSPsyc (MS with psychiatric comorbidities), MS‐Comb (MS with neurological and psychiatric comorbidities)	NA	MDS Score of 2–4 represents mild cognitive impairment, 5−8 represents moderately severe impairment, and 9–10 very severe impairment/ comparison of the proportion of cognitively impaired/improved participants between baseline and follow‐up	NA	MDS‐Cog score at admission predicted MDS‐Cog scores after 1 year.
Demakis et al. (2009)	United States	Short‐and long‐term memory, orientation, and communication pattern	3	924 PwMS: 72% female and 28% male	57.5 (13.5)	MS (MS without psychiatric or neurological comorbidities), MS‐Neuro (MS with neurological comorbidities), MSPsyc (MS with psychiatric comorbidities), MS‐Comb (MS with neurological and psychiatric comorbidities)	NA	MDS Score of 2–4 represent mild cognitive impairment, 5 −8 represent moderately severe impairment and 9–10 very severe impairment/ comparison of the mean MDS score in participants between baseline and follow‐up	NA	The MS‐Neuro and the MS‐Comb groups had more cognitive decline compared to the MS‐Psyc and the MS groups
Eijlers et al. (2018)	The Netherlands	Executive functioning, verbal memory, verbal fluency, Information processing speed, visuospatial memory, attention, working memory	5	294: 234 PwMS –32% male and 68% female, 60 HC –29% male and 71% female)	PwMS: 47.61 (11.02) and HC: 46.45 (9.91)	181 RRMS, 33 SPMS and 20 PPMS.	14.77 (8.43)	< –1.5 SD on ≥ 2 cognitive domains (study controls as norms) / comparison of the proportion of cognitively impaired/improved participants between baseline and follow‐up	Median (range): 3 (0, 8)	Statistically significant cognitive decline was observed in all MS phenotypes but was three times faster in progressive MS patients compared to RRMS patients.
Fuchs et al. (2020)	United States	Processing speed	16	531 PwMS: 22.2% male and 77.8% female, 455 RRMS, 76 PMS	RRMS: 43.4 (9.3) PMS: 50.8 (9.0)	455 RRMS, 76 PMS	Not specified	SDMT age, sex, and education‐corrected z‐scores/SD that is < 0 and > −1.5 were considered as mild impairment and ⩽−1.5 considered as severe impairment/Individual subject trajectories shown with slopes	RRMS—median (IQR): 2.5 (1.5, 3.5) PMS—median (IQR): 5.5 (3.5, 6.0)	A decline of 0.22 SDMT raw score per year was observed in PwMS (statistically significant). Higher trait Conscientiousness, being well‐organized and goal driven was associated with a slower decline in SDMT longitudinally
Healy et al. (2021)	United States	NA	1+ (378 days (median time))	680 PwMS: 171 males and 509 females	42.4 (10.8)	Not defined	9.0 (8.1)	Longitudinal cluster trajectory of SDMT/ trajectories shown in a graph	1.7 (1.7)	PwMS who had lower than average SDMT score at baseline had greater worsening of SDMT score compared to PwMS who had higher than average score
Heled et al. (2021)	Israel	Executive functions, memory, visuospatial perception, verbal functions, attention, information processing speed	8	183 PwMS: 158 males and 25 females	Assessment intervals: 2–3 years: 38.48 (11.11), 4–5 years: 38.81 (12.38), 6–8 years: 38.48 (12.41)	All RRMS	Assessment intervals: 2–3 years: 7.58 (7.21), 4–5 years: 8.68 (8.38), 6–8 years: 6.08 (6.86)	Higher score represented better performance/ comparison of means of participant scores between baseline and follow‐up	Assessment intervals: 2–3 years: 1.97 (1.61), 4–5 years: 2.11 (1.57), 6–8 years: 1.92 (1.37)	There was an observed decline in executive functions, attention, and information processing speed (IPS) between the two‐time points of assessment.
Hoogervorst et al. (2002)	The Netherlands	Components of Multiple Sclerosis Functional Composite (MSFC)	1	120 PwMS: 37% male and 63% female; 40 RRMS, 37 SPMS and 43 PPMS	47.1 years (12.1 years)	40 RRMS, 37 SPMS and 43 PPMS	NA	Higher MSFC score represented better performance/ MSFC score between baseline and follow‐up	Median (IQR)—RRMS: 3.5 (2.0, 4.0) SPMS: 5.5 (4.0, 6.5) PPMS: 6.5 (4.0, 7.5)	MSFC had the highest correlation with cognitive function
Hughes et al. (2015)	United States	Perceived cognitive impairment	3	234 PwMS: 35 males and 199 females	53.83 (10.54)	136 RRMS, 58 SPMS, 28 PPMS and 12 PRMS	15.88 (9.66)	Higher score represented better performance/ comparison of participants’ mean score between baseline and follow‐up	Range: 4.5–6.5 in 47.4% of the participants	There was a strong correlation between the University of Washington Self‐Efficacy Scale (UWSES) and ACGC and ACEF at baseline and follow‐up
Hughes et al. (2018)	United States	Perceived cognitive impairment	1	163 PwMS: 21 males and 142 females	52.2 (10.1)	91 RRMS, 34 SPMS, 28 PPMS and 8 PRMS and 2 not reported	12.0 (9.0)	A higher score represented better performance	NA	Self‐reported Sleep problems are associated with perceived cognitive dysfunction
Jakimovski et al. (2020)	United States	Processing speed, verbal learning and memory, visuospatial learning, and memory	5	199: 127 PwMS (11 males and 89 females), 20 CIS (3 males and 17 females) and 52 HC (12 males and 38 females)	PwMS: 48.4 (10.9) CIS: 42.6 (10.7) HC: 43.8 (15.4)	127 PwMS: 85 RRMS, 42 PMS	PwMS: 16.3 (10.4) CIS: 5.1 (5.7)	< −1.5 SD on ≥ 1 cognitive test (study controls as norms)	(Median (IQR)) PwMS: 2.5 (1.5, 5.5) CIS: 1.5 (1.0, 1.5)	Baseline higher serum neurofilament light chain level predicted reduced cognitive processing speed
Johansson et al. (2020)	Sweden	Processing speed	10	264 PwMS: 85 males and 179 females	71% were more than 41 years	149 RRMS, 115 PMS	> 10 (10 years follow‐up)	< 32 correct responses at baseline and follow‐up	54% had a score between 0–3.5	SDMT score of ⩾32 correct responses predicted sustained participation in social/lifestyle activities
Johnen et al. (2019)	Germany	Processing speed, divided attention and working memory	1	1123 PwMS and CIS: 348 males and 775 females	34.12 (9.67)	622 RRMS and 501 CIS	0.57 (0.61) (Years since symptom onset)	< –2 SD on ≥ 2 cognitive tests (population controls as norms)/ comparison of the proportion of cognitively impaired/improved participants between baseline and follow‐up	1.49 (0.99)	Baseline characteristics and initiation of treatment with disease‐modifying therapies did not predict cognitive changes within the follow‐up period
Jonsson et al. (2006)	Denmark	Verbal Intelligence, attentional Control, mental processing speed, visuospatial memory, problem‐solving, visual organization and naming	5	155: 80 PwMS (19 males 61 females) and 75 HC	Mean and (range): 35 (20–59)	75 RRMS, 3 PPMS, 2 SPMS	Months: 7 (1, 24)	< −1.5 SD on a cognitive test (study controls as norms) / comparison of the proportion of cognitively impaired/improved participants between baseline and follow‐up	Mean (range): 2.7 (0, 6)	Of all the measured domains only, Visual Organization had a significant linear deterioration over 3 years
Lopez‐Gongora et al. (2015)	Spain	Verbal memory acquisition and delayed recall, visual memory acquisition and delayed recall, sustained and complex attention, information processing speed, working memory, verbal fluency and executive function	1	294: 237 PwMS (80 males and 157 females) and 57 HC (13 males and 44 females)	PwMS: 38.5 (10.2) and HC: 40.5 (9.4)	All RRMS	7.4 (7.1)	< −1.5 SD on ≥ 2 cognitive tests (study controls as norms) / comparison of the proportion of cognitively impaired/improved participants between baseline and follow‐up	Median (range): 1.5 (0, 6)	PwMS with cognitive impairment increases from 27.6% at baseline to 31.6% at follow‐up.
McKay et al. (2019)	Sweden	Information processing speed	3+	5704 PwMS (1689 males and 4015 females): 5404 adult‐onset MS and 300 pediatric onset MS	Adult onset, median (IQR): 38.3 (31.4, 45.2) pediatric onset, median (IQR): 25.6 (21.0, 31.7)	99% of Pediatric onset and 97.6% of adult onset were RRMS	Adult onset: 5.2 (1.7‐11.0) Pediatric onset: 10.2 (5.3–15.7)	< −1.5 SD below the normative mean/ trajectory plotted in a graph with the mean score for each group	NA	SDMT score statistically significantly declined faster for pediatric onset than adult‐onset MS.
Motyl et al. (2021)	Czech Republic	Processing speed	1	1091 PwMS: (332 males 759 females)	38.4 (9.0)	90.8% were RRMS and 9.2% were SPMS	9.9 (7.3)	< −1.5 SD on a cognitive test (study controls as norms) / comparison of the proportion of cognitively impaired/improved participants between baseline and follow‐up	All groups: Median, (IQR) 2.0 (1.5, 3.5)	Only 4% of PwMS experienced isolated cognitive decline and this occurred without a corresponding neurological clinical activity
Raimo et al. (2020)	Italy	Global cognitive functioning, verbal learning (immediate and delayed recall), visuospatial memory (delayed recall), short‐term (verbal) memory, visuospatial functions, executive functions, abstraction, mental flexibility, motor Programming, sensitivity to interference by conflicting instructions test, Inhibitory control, environmental autonomy), executive domain	2+	100 PwMS (gender not specified)	Not specified	Not defined	Not defined	A higher score on Cognitive Impairment Index (CII) represents a poorer cognitive function. Higher CII depends on a higher number of SD from the control mean. (Population controls as norms)/ Comparison of mean score between participants from baseline to follow‐up	NA	A higher degree of apathy at baseline predicted cognitive changes (CII) at follow‐up.
Roy et al. (2018)	United States	NA	5	182 (82 PwMS (17 males and 65 females) and 100 HC (29 males and 71 females))	PwMS: 45.96 (8.13), HC: 45.42 (11.68)	Not defined	9.56 (6.61)	0.8 reliable change indices (decline) in at least one BICAMS measure/graph of trajectory between baseline and follow‐up	Median, (range): 2.75, (0.00, 6.50)	Personality changes were observed in PwMS with cognitive decline
Strober et al. (2019)	United States	Cognition	SDMT: 2 PASAT: 5	1512 PwMS (441 males and 1071 females): Both SDMT and PASAT	36.5 (9.79)	Both SDMT and PASAT: All RRMS	3.6 (4.71)	≤ 4‐point worsening of SDMT	Both SDMT and PASAT: 2.5 (1.23)	A ≤4‐ point worsening of SDMT was the strongest predictor of worsening in the Physical Component Summary
Uher et al. (2017)	Czech Republic	Processing speed, memory (visual modality), memory (auditory sphere)	2	1052 PwMS: 770 Cognitively preserved (29% male, 71% female) and 282 Cognitively impaired (33% male, 67% female)	Cognitively preserved: 37.6 (8.8) Cognitively impaired: 39.6 (8.8)	Not defined	Cognitively preserved: 8.8 (6.7) Cognitively impaired: 11.6 (7.6)	< –1.5 SD on a cognitive test (study controls as norms) / comparison of the proportion of cognitively impaired/improved participants between baseline and follow‐up	Cognitively preserved: 2.2 (1.1) Cognitively impaired: 3.4 (1.4)	Low brain parenchymal fraction and high T2 lesion volume were associated with an increased risk of cognitive decline over the follow‐up period
Wallach et al. (2020)	United States	NA	Mean: 1.8+ years	616 PwMS (217 males and 399 females): Administered SDMT	SDMT decline: 14.3 (13.2, 16.6) SDMT stable or improve: 13.3 (11.9, 15.8)	Not defined	SDMT decline: 2.7 [0.6, 3.8] SDMT stable or improve: 2.9 [0.8, 4.2]	< –1 SD on a cognitive test (SDMT) over time (population controls as norms)/ comparison of the proportion of cognitively impaired/improved participants between baseline and follow‐up	Score: < 2.0 (274 PwMS) ⩾2.0 (109 PwMS)	14.1% of PwMS had SDMT decline at follow‐up predicted by older age and male gender
Wu et al. (2020)	Australia	NA	2.5	1958 PwMS (367 males, 1591 females): 1313 RRMS, 214 SPMS, 170 PPMS and 253 unsure	45.0 (10.2)	RRMS, SPMS and PPMS	< 5 years: 904; 5‐ 10 years: 450 and > 10 years: 602	Perceived cognitive impairment: cognitive function sub‐score ≤66.7 points/ comparison of the proportion of cognitively impaired/improved participants between baseline and follow‐up	NA	Persistent Perceived cognitive impairment was associated with a significantly higher risk of sexual dysfunction at follow‐up.
Ytterberg et al. (2008)	Sweden	1. NA	2	200 PwMS (65 males and 135 females)	47 (12)	119 RRMS, 73 SPMS and 8 PMS	14 (10)	Not defined/ mean score at baseline and follow‐up	Range: 0 (1 PwMS), 1 −3.5 (122 PwMS), 4–5.5 (34 PwMS) and 6–9.5 (43 PwMS)	A higher mean number of correct answers in SDMT was observed in PwMS in the EDSS category 1–3.5 compared to PwMS with EDSS 4 and above

RRMS: relapsing‐remitting multiple sclerosis; PPMS: primary progressive multiple sclerosis; SPMS: secondary progressive multiple sclerosis; PwMS: people living with multiple sclerosis. SDMT: Symbol Digit Modalities Test; PASAT: Paced Auditory Serial Addition Test; SD: standard deviation; NA: not available; IQR: interquartile range; HC: healthy control; EDSS: Expanded Disability Status Scale; BICAMS: Brief International Cognitive Assessment for Multiple Sclerosis; ACGC: applied cognition—general concerns. ACEF: applied cognition—executive function; MSFC: multiple sclerosis functional composite.

**TABLE 2:** Characteristics of studies measuring change in cognitive function in people living with multiple sclerosis after intervention.

Author and year	Location	Cognitive domain	The maximum gap between the measurement of cognition (years)	Study population	Age (at baseline) Mean (SD) years	Patient group (RRMS, PPMS, SPMS) at baseline	MS duration (mean) at baseline: Mean (SD) years	Definition of cognitive impairment (or improvement)/ Change in cognitive function	EDSS score (mean) at baseline: Mean (SD)	Findings about cognitive function	Any intervention/ medication
Amato et al. (2010)	Italy	Verbal memory acquisition and delayed recall, visual memory acquisition and delayed recall, attention, concentration, and speed of information processing, verbal fluency on semantic stimulus, and Cognitive control and inhibition	3	105: 49 PwMS (11 males and 38 females) and 56 HC (20 males and 36 females)	PwMS: 36.9 (8.9) and HC: 38.1 (9.0)	All Relapsing‐remitting	2.9 (1.7)	< −1.5 SD on ≥ 2 cognitive tests (study controls as norms)/ Comparison of mean scores for each test among participants between baseline and follow‐up	1.7 (0.7)	Moderate cognitive impairment at baseline predicted more cognitive decline later at follow‐up	Therapy with interferon β (IFNB)‐1b.
Benedict et al. (2021)	United States	Processing speed, working memory, episodic memory (visuospatial memory)	2	1651 PwMS (659 males and 992 females)	48.0 (7.9)	All secondary progressive (SP) MS	12.6 (7.8)	4‐point sustained increase (improvement) or decrease (deterioration) in SDMT. Impairment is defined as SDMT ≥2 SD below the mean of study controls/mean change in SDMT score from baseline to follow‐up	Median: 6.0	Patients treated with siponimod had significant improvement in SDMT	Siponimod versus placebo
Benedict et al. (2018)	United States	Cognitive processing speed	2+ (144 weeks)	1841 PwMS (919 daclizumab β and 922 IM Interferon β‐1a) Sex ratio not defined.	Range: 18, 55 years	All RRMS	Not defined	3‐point or 4‐point change in SDMT score from baseline to follow‐up/ mean change in SDMT score from baseline to follow‐up	Range: 0, 5.0	Clinically meaningful improvement (an increase from baseline of ⩾3 points) in cognitive function (SDMT) observed from daclizumab β compared to intramuscular interferon (IFN) β‐1a	Daclizumab β compared to intramuscular interferon (IFN) β‐1a
Benesova & Tvaroh (2017)	Czech Republic	NA	2	300 PwMS: 300 relapsing‐remitting MS (RRMS) patients (102 males and 198 females) but 272 analyzed	36.33 (10.04)	All relapsing‐remitting MS patients	5+ (61.6 months)	Increase in mean PASAT score from baseline to follow‐up (not statistically significant)/ proportion of participants with increased, stable or decreased PASAT at baseline and follow‐up	2.85 (1.10)	Improvement in cognition (PASAT score) in patients taking subcutaneous IFN β‐a (change in PASAT compared to stable PASAT score)	All PwMS were on different doses of subcutaneous IFN β‐1a
Chan et al. (2017)	United Kingdom	Premorbid Intelligence Quotient (IQ), intellectual function, verbal IQ, nonverbal IQ, verbal intelligence, abstract verbal reasoning, spatial perception and visuomotor skills, nonverbal abstract reasoning, semantic memory, verbal episodic memory, verbal episodic memory, visuoperceptual functioning, nonverbal episodic memory, nonverbal episodic memory, spatial perception, executive functions, information processing, attention, and working memory	2	140 PwMS: 70 on Simvastatin and 70 on placebo	51·3 (6·9)	All secondary progressive multiple sclerosis	21·2 (8·6)	< −1.5 SD on a cognitive test (study controls as norms) /mean change in test scores from baseline to follow‐up	5·8 (0·8)	There was a significant decline in verbal memory and nonverbal memory in all PwMS. FAB score higher in the Simvastatin group compared to the placebo group	Simvastatin versus placebo
Cinar et al. (2017)	Turkey	Verbal learning and memory, visual‐spatial memory	1	263: 161 PwMS (51 males and 110 females) and 102 HC (34 males and 68 females)	PwMS: 30.4 (11.2) HC: 31.5 (14.3)	All RRMS	2.02 (2.6)	< −1.5 SD on a cognitive test (study controls as norms)/mean test scores at baseline and follow‐up	Score: < 5.5	Cognitive test scores were significantly poorer in the PwMS The proportion of cognitively impaired PwMS reduced after a year of the administration of glatiramer acetate and β‐interferon	Glatiramer acetate and β‐interferon
Comi et al. (2017)	Italy	Executive functions	1.5 (18 months)	157 PwMS (106 for fingolimod and 51 for Interferon B)	Fingolimod: 40.23 (9.09) and Interferon B: 37.64 (9.29)	All RRMS	Fingolimod: 4.97 (6.67) and Interferon B: 4.71 (6.47)	< −1.5 SD on ≥ 1 cognitive test (population controls as norms based on age and gender)/mean change in test scores between baseline and follow‐up	Fingolimod: 2.78 (1.34) and Interferon B: 2.09 (1.05)	Both treatment groups showed significant improvement in cognitive function. No significant differences were observed between the treatment groups	Fingolimod and interferon β‐1b
DeLuca et al. (2021)	United States	Processing speed	1	830 PwMS: 418 took ozanimod and 412 interferon β‐1a (63.3% and 67% in each group were females, respectively)	Ozanimod: 34.8 years and Interferon β‐1a: 35.9 years	All RRMS	6.9	4‐point or greater increase (SDMT improved) or decrease (worsened) in SDMT score from baseline to follow‐up was observed as improvement or worsening respectively/ Proportion of participants with improved or worsened SDMT score at baseline and follow‐up	PwMS on Ozanimod: SDMT improved: 2.5 (1.0), worsened: 2.5 (1.2) and PwMS Interferon β‐1a: SDMT improved 2.5 (1.2), worsened: 2.7 (1.2)	Ozanimod significantly enhanced cognitive scores compared with Interferon β‐1a	Ozanimod compared to Interferon β‐1a
De Giglio et al. (2016)	Italy	Verbal memory acquisition and delayed recall, visuospatial memory acquisition and delayed recall, sustained attention, Concentration, processing speed, and verbal fluency on semantic stimulus.	2	142 PwMS (All female)	Subcutaneous IFN‐b‐1a: 30.4 (7.0) Subcutaneous IFN‐b‐1a and ethinyl estradiol 20 μg: 29.1 (6.4) and Subcutaneous IFN‐b‐1a and ethinyl estradiol 40 μg: 30.6 (5.9)	All RRMS	Subcutaneous IFN‐b‐1a: 4.2 (4.5) subcutaneous IFN‐b‐1a and ethinyl estradiol 20 μg: 3.3 (3.0) and Subcutaneous IFN‐b‐1a and ethinyl estradiol 40 μg: 3.5 (3.9)	< –1 SD on ≥ 2 cognitive tests (population controls as norms)/proportion of participants with cognitive impairment at baseline and follow‐up	Subcutaneous IFN‐b‐1a: 1.7 (0.7) subcutaneous IFN‐b‐1a and ethinyl estradiol 20 μg: 1.8 (0.9) and Subcutaneous IFN‐b‐1a and ethinyl estradiol 40 μg: 1.6 (1.0)	The proportion of cognitively impaired PwMS less in subcutaneous IFN‐b‐1a and ethinyl estradiol 40 μg groups	Subcutaneous IFN‐b‐1a and ethinyl estradiol
Fischer et al. (2000)	United States	Information processing, visual learning/recall, verbal learning/recall, visuospatial abilities, problem‐solving, visual scanning, planning/sequencing, deductive reasoning, general verbal abilities, attention span, Information processing, general verbal abilities	2	166 PwMS (128 females, 38 males and 83 Interferonβ‐1a, 83 placebo)	Interferon β‐1a: 36.1 (6.4) Placebo: 36.2 (6.8)	All RRMS	Interferon β‐1a: 6.7 (5.7) Placebo: 6.4 (5.1)	Having a score that is less than 0.5 SD relative to week 0 (baseline) score (after adjustment)/mean z score at baseline and follow‐up	Interferon β‐1a: 2.3 (0.8) Placebo: 2.4 (0.9)	RRMS patients performed better cognitively (information processing and learning/recent memory measures, visuospatial abilities, and executive functions) while on Interferon β‐1a compared to those on a placebo	Interferon β‐1a versus placebo
Iaffaldano et al. (2012)	Italy	Verbal memory acquisition and delayed recall, visual memory acquisition and delayed recall, attention, concentration and speed of information processing, verbal fluency on semantic stimulus, frontal lobe executive functions	Main group: 1 year. Smaller group: 2 years	100 PwMS (main group): 28 males and 72 females	34.55 (9.25) (main group)	All RRMS	11.09 (7.52)	< 2 SD on ≥ 3 cognitive tests (population controls as norms)/proportion of participants with Cognitive Impairment at baseline and follow‐up	3.66 (1.14)	Cognitively impaired participants decreased significantly between baseline and follow‐up (1 year)	Natalizumab
Koch et al. (2021)	Canada	NA	2	889 PwMS: 339 males and 550 females	47.2 years (7.6)	All SPMS	Has had SPMS for two or more years	Four‐point change in SDMT and PASAT score between baseline and follow‐up	(Median (IQR)): 6.0 (5.0, 6.5)	There was an increased improvement in SDMT score in about 75% of the participants from baseline to 104 weeks.	Natalizumab
Lincoln et al. (2020)	United Kingdom	Total recall and delayed recall, total recall and delayed recall, information processing speed, easy total and hard total, word fluency, memory function, attention, and executive abilities	1	449 PwMS (123 males and 326 females)	49 (SD 9.9)	291 RRMS, 46 PPMS and 112 SPMS	11.7 (8.4)	< −1 SD on a cognitive test (study controls as norms)/mean test scores at baseline and at follow‐up	NA	No long‐term benefits on quality of life were observed in MS patients after the cognitive rehabilitation program	Cognitive rehabilitation versus usual care
Patti et al. (2010)	Italy	NA	3	459 PwMS: 236 on sc IFNb‐1a (44 μg) and 223 on sc IFNb‐1a (22 μg)	33.3 (8.13) years	All RRMS	3.8 (4.47)	< −1 SD on a cognitive test (population controls as norms)/proportion of cognitively impaired participants at baseline and follow‐up	1.8 (1.0)	For PwMS on 44 μg, cognitive impairment remained stable at 15.2% from baseline to follow‐up. For PwMS on 22 μg cognitive impairment increased from 23.5% to 24.8%	subcutaneous IFNb‐1b (Interferon‐1b)
Patti et al. (2013)	Italy	NA	5+	265 PwMS (99 males and 166 females)	39 (8.2)	All RRMS	8 (4.4)	< −1 SD on ≥ 3 cognitive tests (population controls as norms)/proportion of cognitively impaired participants at baseline and follow‐up	NA	The proportion with cognitive impairment remained so at 3 years and at 5 years however men were more affected.	Subcutaneous (sc) interferon (IFNb‐1b) 44 or 22 mg
Perumal et al. (2019)	United States	NA	2	222 PwMS (61 males and 161 females)	34.0 (8.97)	All RRMS	1.6 (0.77)	Higher scores indicate better cognitive function/ change in SDMT score between baseline and follow‐up	2.0 (1.13)	PwMS had a significant increase in SDMT score between baseline and follow‐up (2 years)	Natalizumab
Rudick et al. (2009)	United States	NA	2	942 PwMS (627 on natalizumab and 315 on placebo (AFFIRM))	AFFIRM^‡^: Natalizumab 35.6 (8.5) Placebo 36.7 (7.8)	AFFIRM: All RRMS	AFFIRM^‡^: Natalizumab—median (range) 5.0 (0, 34); Placebo—median (range) 6.0 (0, 33)	A ≥ 20% worsening of MSFC† score (PASAT‐3) sustained for 3 months at follow‐up compared to the baseline score/ proportion of participants that had the worsening of MSFC from baseline to follow‐up	AFFIRM^‡^: Natalizumab 2.3 (1.2) Placebo 2.3 (1.2)	A ≥ 20% worsening of MSFC score was significantly associated with EDSS change.	Natalizumab and placebo
Schwartz et al. (1996)	United States	Verbal and spatial memory, complex attention, verbal fluency, executive function/cognitive flexibility, complex attention, and sequencing	1	130 PwMS (27.4% male and 72.6% female)	43.2 (8.93)	41.5% RRMS and 58.5%Chronic Progressive	8.26 (6.71)	Number of cognitive tests < −1 SD, ← 2, ← 3 of normative mean/ mean *T* score at baseline and follow‐up	4.74 (1.74)	The interventions had no differential effect on neuropsychological function over time	Two psychosocial interventions (not explained but reference provided)
Schwid et al. (2007)	United States	Verbal learning and memory, visuospatial memory and learning, attention, information processing, and working memory, verbal fluency	10+	153 PwMS (gender proportion not specified)	34.6 (6.1)	All RRMS	6.9 (5.2)	A 0.5 SD reduction in test scores compared to the norm in a cognitive test/the proportion of the cognitively impaired in baseline and follow‐up	2.6 (1.2)	Cognitive test score changes in 2 years predicted 10‐year changes	Glatiramer acetate and placebo
Stephenson et al. (2012)	United States	NA	1	333 PwMS (21.9% male and 78.1% female)	46.8 (10.4)	Not defined	10.6 (7.9)	One point change in scale (MS‐COG) between baseline and follow‐up	NA	Natalizumab reduced the effect of MS on cognitive function. 65% to 69% of PwMS showed improvement at follow‐up compared to baseline.	Natalizumab
Weinstein et al. (1999)	United States	Verbal learning and recall, visuospatial learning and delayed recall, perceptual processing and attention, information processing speed and sustained attention, semantic retrieval and production	2	251 PwMS (67 males and 184 females)	Glatiramer: 34.6 (6.0) Placebo: 34.3 (6.5)	All RRMS	Glatiramer: 7.3 (4.9) Placebo: 6.6 (5.1)	< −2 SD on a cognitive test (population controls as norms/mean test scores at baseline and follow‐up	Glatiramer: 2.8 (1.2) Placebo: 2.4 (1.3)	Mean cognitive test function scores were higher at follow‐up (1 and 2 years) compared to the baseline	Glatiramer and Placebo
Weinstock‐Guttman et al. (2012)	United States	NA	2	856 PwMS: 574 on natalizumab group and 282 on placebo (AFFIRM^‡^). 887 PwMS: 459 on natalizumab and IFNb‐1a and 428 on placebo and IFNb‐1a group (SENTINEL^§^).	Not specified	All RRMS	Not specified	Cognitive decline progression: 0.5 SD worsening in PASAT‐3 score observed for 12 weeks	Range: 0.0−5.0	Natalizumab reduced the risk of observed progression of cognitive deficits by 43% compared with the placebo	Intravenous natalizumab 300 mg, intramuscular (IM) IFNb‐1a 30 lg and placebo

^†^ MSFC: multiple sclerosis functional composite.

^‡^ AFFIRM: natalizumab safety and efficacy in relapsing‐remitting multiple sclerosis.

^§^ SENTINEL: safety and efficacy of natalizumab in combination with interferon β‐1a in patients with relapsing‐remitting multiple sclerosis.

Abbreviations: RRMS: relapsing‐remitting multiple sclerosis; PPMS: primary progressive multiple sclerosis; SPMS: secondary progressive multiple sclerosis; PwMS: people living with multiple sclerosis; SDMT: Symbol Digit Modalities Test; PASAT: Paced Auditory Serial Addition Test; SD: standard deviation; NA: not available; IQR: interquartile range; HC: healthy control; EDSS: Expanded Disability Status Scale; MS‐COG: Medical Outcomes Scale‐Cognitive Functioning; FAB: Frontal Assessment Battery.

### 3.1 Change in cognitive function

Most observational studies focused predominantly on cognitive impairment at baseline and follow‐up or cognitive decline. However, in some studies (interventional) the focus was on improvement in cognitive function or both improvement and decline (from baseline to follow‐up) (Benedict et al., 2018; Benedict et al., 2021; DeLuca et al., 2021). There was significant heterogeneity in the definition of cognitive impairment in studies that used a cognitive test battery or a singular cognitive test. Most papers that used a test battery defined impairment as failure in 1 or more tests with a cut‐off score of < −1.5 SD from the mean (normative or study healthy control). However, there was heterogeneity in the use of a cut‐off score with −2.0, −1.5, and −1.0 SD from the mean (normative or study healthy control), and heterogeneity in the number of qualifying failed tests (1 to 5 or more tests), to define “impairment.” Decline or improvement was shown by comparing the percentage/proportion of cognitively impaired to improved participants between baseline and follow‐up (Amato & Ponziani, 1998; Andravizou et al., 2020; Chruzander et al., 2014; Johnen et al., 2019) and reliable change index was used to report meaningful change as it corrects for measurement error and practice effects (Andravizou et al., 2020). For some papers that used one cognitive test, a 10% or 20% change in mean score (Bsteh et al., 2019) or a 4‐point change (SDMT mostly) in the score (Benedict et al., 2018; DeLuca et al., 2021; Koch et al., 2021) were used.

### 3.2 The cognitive function domains measured, and the tests and batteries used

Visual learning and memory (21/57) and verbal learning and memory (20/57) were the most frequently measured domains when a battery was used (Table 3). Information processing speed and complex attention (34/57) was the most measured domain when cumulatively a single test or a battery was used. Rao's BRNB and BICAMS were the predominant batteries used in the measurement of change in cognitive function in PwMS (19/57). Other studies used Randt's memory battery, Birt Memory and Information Processing Battery (BMIPB), and Visual Object and Space Perception (VOSP) battery (Chan et al., 2017). In some cases, it was a combination of cognitive tests from different batteries that were used (Jonsson et al., 2006).

**TABLE 3:** Cognitive function domains and the cognitive tests and batteries that were used in included studies.

Domains	Cognitive test (Cognitive test battery†)
Premorbid intelligence	National Adult Reading Test (Chan et al., 2017)
Intellectual functioning	Wechsler Abbreviated Scale of Intelligence (WASI; Full‐Scale IQ, Performance IQ, and Verbal IQ) (Chan et al., 2017) Wechsler Adult Intelligence Scale–Revised (WAIS‐R) (Fischer et al., 2000)
Short‐term storage capacity or attention span	Digits Forward (Jonsson et al., 2006) Wechsler Memory Scale–Revised (WMS‐R) (Fischer et al., 2000)
Complex attention—information processing speed and visual tracking	Symbol Digit Modalities Test (single test and in a battery) (RBRB†) (Amato et al., 2010; Amato et al., 2010; Benedict et al., 2018; Benedict et al., 2021; Borghi et al., 2016; Bsteh et al., 2019; Chruzander et al., 2014; Crielaard et al., 2019; De Giglio et al., 2016; de Groot et al., 2009; DeLuca et al., 2021; Eijlers et al., 2018; Fuchs et al., 2020; Healy et al., 2021; Iaffaldano et al., 2012; Jakimovski et al., 2020; Johansson et al., 2020; Jonsson et al., 2006; Lincoln et al., 2020; Lopez‐Gongora et al., 2015; McKay et al., 2019; Motyl et al., 2021; Patti et al., 2010; Perumal et al., 2019; Roy et al., 2018; Schwartz et al., 1996; Schwid et al., 2007; Uher et al., 2017; Wallach et al., 2020; Weinstein et al., 1999; Ytterberg et al., 2008) The Trail Making Test—Part A (Raimo et al., 2020; Schwartz et al., 1996) California Computerized Assessment Package (CALCAP) Sequential reaction time (Fischer et al., 2000) Memory Comparison Test (Eijlers et al., 2018)
Working memory	Paced Auditory Serial Addition Test (single test and in a battery) (Amato et al., 2010; Benedict et al., 2021; Benesova & Tvaroh, 2017; Borghi et al., 2016; Bosma et al., 2010; Chan et al., 2017; Comi et al., 2017; De Giglio et al., 2016; de Groot et al., 2009; De Meijer et al., 2018; Fischer et al., 2000; Hoogervorst et al., 2002; Iaffaldano et al., 2012; Johnen et al., 2019; Koch et al., 2021; Lincoln et al., 2020; Lopez‐Gongora et al., 2015; Patti et al., 2010; Rudick et al., 2009; Schwartz et al., 1996; Schwid et al., 2007; Strober et al., 2019; Weinstein et al., 1999; Weinstock‐Guttman et al., 2012) Serial Seven Subtraction Test (Jonsson et al., 2006) Arithmetical Operations (Jonsson et al., 2006) Digits Backward (Jonsson et al., 2006)
Visual scanning	Visual search number of trials (Fischer et al., 2000) Mesulam Cancellation Test (Jonsson et al., 2006)
Visual perception and organization	Street Gestalt Completion Test (Jonsson et al., 2006)
Visual construction	Rey– Osterrieth Complex Figure Test (ROCF)—Copy (Jonsson et al., 2006; Raimo et al., 2020) (see also Executive Function—planning) Block Design (Wechsler Abbreviated Scale of Intelligence) (Chan et al., 2017) Figure Copying (BIRT† Memory and Information Processing Battery) (Chan et al., 2017) Clock Drawing Test (CDT) (Raimo et al., 2020)
Language	The Aachener Aphasie Test (Amato et al., 2010) Vocabulary (WASI/WAIS) (Chan et al., 2017; Jonsson et al., 2006) Boston Naming (split half version, odd‐numbered tasks) (Jonsson et al., 2006) Graded Naming Test (Chan et al., 2017)
Verbal learning and memory	Selective Reminding Test (RBRB†) (Amato et al., 2010; Amato et al., 2010; Borghi et al., 2016; Comi et al., 2017; De Giglio et al., 2016; de Groot et al., 2009; Eijlers et al., 2018; Iaffaldano et al., 2012; Jonsson et al., 2006; Lincoln et al., 2020; Lopez‐Gongora et al., 2015; Patti et al., 2010; Schwartz et al., 1996; Schwid et al., 2007; Weinstein et al., 1999) Story: Immediate and Delayed Recall (BIRT† Memory and Information Processing Battery) (Chan et al., 2017) The California Verbal Learning Test‐II (BICAMS†) (Cinar et al., 2017; Fischer et al., 2000; Jakimovski et al., 2020; Uher et al., 2017) Word List Learning (2 trials), Interference Word List Learning, and Word List Recall (Raimo et al., 2020) Rey Auditory Verbal Learning Test (RAVLT) (Raimo et al., 2020)
Visual learning and memory	10/36 Spatial Recall Test (SPART) (RBRB†) (Amato et al., 2010; Borghi et al., 2016; Comi et al., 2017; De Giglio et al., 2016; de Groot et al., 2009; Eijlers et al., 2018; Iaffaldano et al., 2012; Jonsson et al., 2006; Lincoln et al., 2020; Lopez‐Gongora et al., 2015; Patti et al., 2010; Schwartz et al., 1996; Schwid et al., 2007; Weinstein et al., 1999) 7/24 Spatial Recall Test (Jonsson et al., 2006) Brief Visuospatial Memory Test—Revised (BICAMS†) (Benedict et al., 2021; Cinar et al., 2017; Jakimovski et al., 2020; Roy et al., 2018; Uher et al., 2017) Rey–Osterrieth Complex Figure Test (ROCF)—Delay Recall (Jonsson et al., 2006; Raimo et al., 2020) Figure Copying: Delayed recall (BIRT† Memory and Information Processing Battery) (Chan et al., 2017)
Cognitive control	Modified Stroop Task, Stroop Test‐interference task (Raimo et al., 2020) The Stroop Test (Amato et al., 2010) Stroop Color Naming Test (simplified version) (Jonsson et al., 2006) Stroop Color‐Word Task (Iaffaldano et al., 2012; Lopez‐Gongora et al., 2015) (Eijlers et al., 2018) (Patti et al., 2010) Inverse Motor Learning Test (IML) (Raimo et al., 2020)
Cognitive flexibility/divided attention	Category Fluency Switch Condition (Raimo et al., 2020) The Trail Making Test—Part B (Schwartz et al., 1996) Trail Making: Part B—Part A (DKEFS†) (Lincoln et al., 2020) Trail Making Test (TMT: B, TMT: B‐A) (Raimo et al., 2020)
Motor performance	Constructional Apraxia Task (CAT) (Raimo et al., 2020)
Executive function—planning	Tower of London Test (Amato et al., 2010; Fischer et al., 2000; Jonsson et al., 2006) Rey—Osterrieth Complex Figure Test (ROCF)—Copy (Raimo et al., 2020) (see also visual construction)
Executive function—fluency	Word List Generation (Amato et al., 2010; Borghi et al., 2016; Comi et al., 2017; De Giglio et al., 2016; de Groot et al., 2009; Eijlers et al., 2018; Iaffaldano et al., 2012; Lopez‐Gongora et al., 2015; Patti et al., 2010; Schwid et al., 2007) Word Fluency Total Score (Lincoln et al., 2020) Controlled Oral Word Association Task (Schwartz et al., 1996) Words starting with letter “s” (Jonsson et al., 2006) Phonological verbal fluency task, and semantic verbal fluency task (Raimo et al., 2020) Animals (Jonsson et al., 2006) Design Fluency (Jonsson et al., 2006) Ruff Figural Fluency Test (Fischer et al., 2000)
Executive function—visual concept formation or abstract reasoning	Raven's Colored Progressive Matrices (RCPM) (Raimo et al., 2020) Raven Progressive Matrices (Jonsson et al., 2006) Matrix Reasoning (WASI/WAIS) (Chan et al., 2017; Lopez‐Gongora et al., 2015) Cube Analysis Task (The Visual Object and Space Perception battery) (Chan et al., 2017)
Executive function—verbal concept formation or abstract reasoning	Similarities (WAIS) (Chan et al., 2017; Jonsson et al., 2006) 20 Qs % good hypothesis Qs (Fischer et al., 2000)
Executive function—set shifting	Free Sorting and Sort Recognition—Sorting Test (DKEFS†) (Comi et al., 2017) The Concept Shifting Test (Eijlers et al., 2018) The Wisconsin Card Sorting Test (WCST) (Fischer et al., 2000; Lopez‐Gongora et al., 2015; Schwartz et al., 1996)
Social cognition	Famous Faces (naming %) (Jonsson et al., 2006)
Multiple domains—global cognition	Blessed Information‐Memory Concentration Test (“Information Test”—personal orientation; “Memory”—personal and nonpersonal knowledge and remembering a name and address; and “Concentration”—saying months of year backward and counting 1–20 forward and backward (Amato & Ponziani, 1998; Amato et al., 1995; Amato et al., 2001) Digit forward, Five items and paired word acquisition (Amato & Ponziani, 1998; Amato et al., 1995; Amato et al., 2001) (Randt's Memory Battery†) Frontal Assessment Battery (Chan et al., 2017) Similarities task, lexical fluency, Luria's motor series, Conflicting instructions test, Go/no go test and Prehension Behavior Test (Frontal Assessment Battery†) (Raimo et al., 2020) Mini‐Mental State Examination Italian version (Raimo et al., 2020) GO‐NOGO Response Inhibition Test, Stroop interference test, catch game, verbal memory, nonverbal memory, staged information processing speed, finger tapping, verbal function, visual‐spatial processing (NeuroTrax (NT; NeuroTrax Corp, Modiin)) (Heled et al., 2021)
Comprehension, expression, social interaction, problem‐solving, and memory	Functional Independence Measure (Beckerman et al., 2013)
Episodic memory	Brief Visuospatial Memory Test—Revised (Benedict et al., 2021)
Short‐ and long‐term memory, orientation, and communication	MDS‐Cognition Scale (11‐point scale) (Demakis & Buchanan, 2010; Demakis et al., 2009)
Self‐reported cognitive function	Applied Cognition—General Concerns and Applied Cognition—Executive Function (Neurological Disorders (NeuroQoL) measurement system) (Hughes et al., 2015), (Applied Cognition–General Concerns Short Form Version 1 (NeuroQoL) measurement system) (Hughes et al., 2018), Medical Outcomes Scale‐Cognitive Functioning (MOS‐Cog) (Stephenson et al., 2012), MS Quality of Life‐54 (MSQOL‐54) (Wu et al., 2020)
No mention of cognitive domain or test	(RBRB†) (Patti et al., 2013)

Abbreviations: RBRB: Rao's Brief Repeatable Battery; BICAMS: Brief International Cognitive Assessment for Multiple Sclerosis; DKEFS: Delis‐Kaplan Executive Function System.

† Cognitive test battery.

Thirty‐nine studies used cognitive test batteries and nineteen studies used individual cognitive tests. Among the studies that used only one cognitive test, most used either the SDMT (13 articles) or Paced Auditory Serial Addition Test (PASAT) (5 articles). Both were additionally reported by some authors to measure attention (sometimes qualified as sustained attention) and/or concentration (Amato et al., 2010; De Giglio et al., 2016; Iaffaldano et al., 2012) Overall, considering both studies that used batteries or a single test, the SDMT was used in the highest number of studies (*n* = 31).

### 3.3 Results related to change in cognitive function from the included papers

Most longitudinal studies without any intervention reported cognitive decline between baseline and follow‐up and some occurred without associated clinical neurological deterioration based on EDSS and relapse (Motyl et al., 2021). Other observed findings include that the trend of cognitive decline in PwMS depended on the associated comorbidities: psychiatric conditions such as depression, bipolar disorder, schizophrenia, or neurological conditions such as Alzheimer's and Parkinson's diseases, or both (Demakis et al., 2009). Cognitive decline in PwMS is associated with poorer work outcomes (Amato & Ponziani, 1998), poorer health‐related quality of life (Chruzander et al., 2014) and personality changes (Raimo et al., 2020). A decline in cognitive function was theorized to occur gradually over time in PwMS as opposed to it occurring in a step‐wise manner (as would occur for example in stroke) (Demakis & Buchanan, 2010; Healy et al., 2021). There was also a greater decline in progressive MS than RRMS (Eijlers et al., 2018), older PwMS, and males with MS (de Groot et al., 2009; Wallach et al., 2020). Pediatric onset (i.e., < 18 years of age) is also associated with greater cognitive decline than adult‐onset MS (McKay et al., 2019). Cognitive dysfunction is also more likely to occur in PwMS with an EDSS of more than 3.5 compared to those with an EDSS of 1 to 3.5 (Ytterberg et al., 2008).

### 3.4 Meta‐analysis of SDMT

**FIGURE 2:**
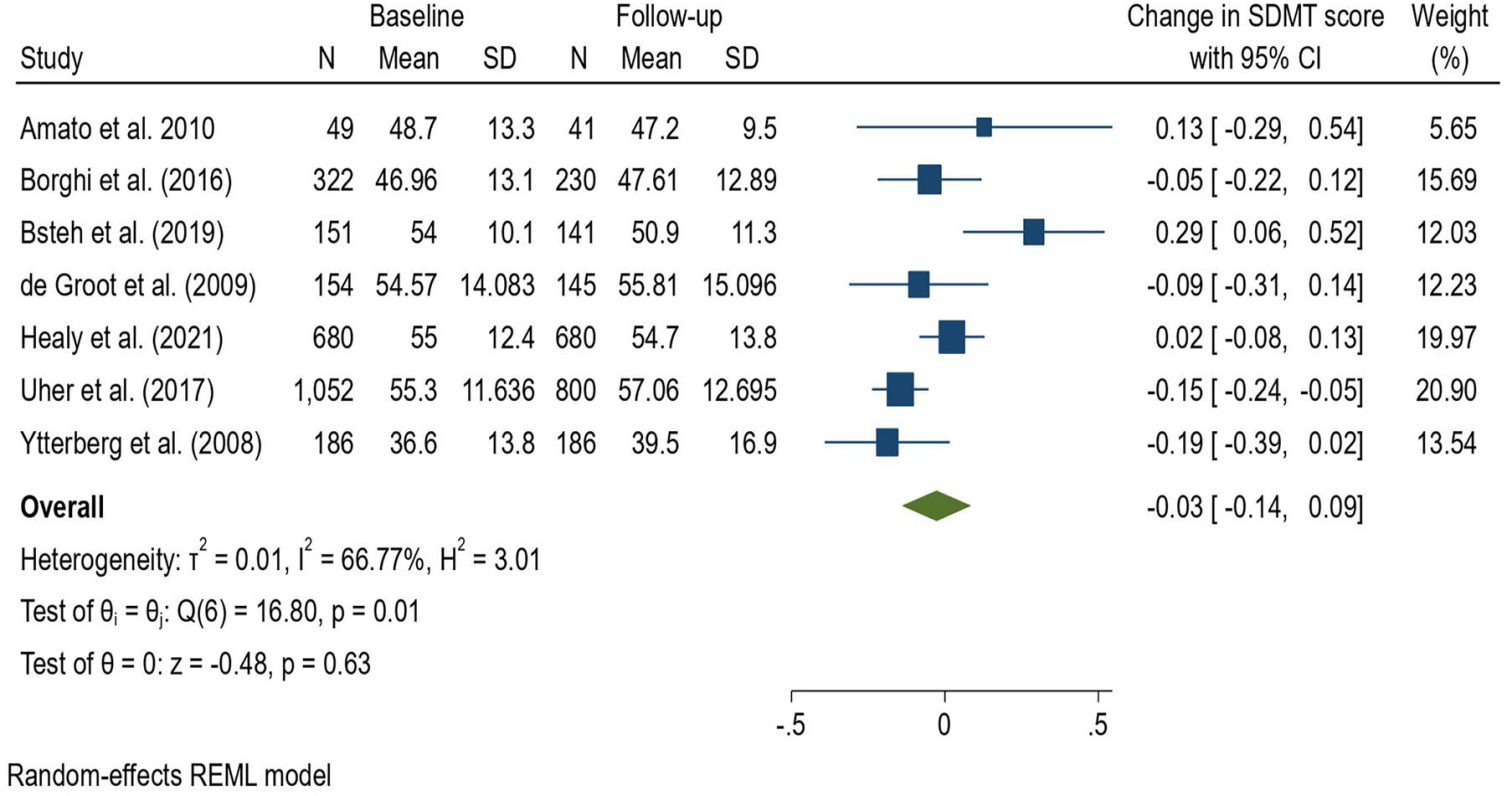
Forest plot of the effect size of change in Symbol Digit Modalities Test between baseline and follow‐up.

In the meta‐analysis of SDMT, 2594 participants were pooled from 7 observational study papers (Figure 2). Baseline and follow‐up measurement intervals of cognitive function in the 7 papers ranged from 1 year to 3 years. The results showed that there was an associated −0.03 (95% CI −0.14, 0.09) decrease in SDMT mean score per year in PwMS, although this was not statistically significant. Sensitivity analysis using linear regression that included the 7 papers did not show any statistically significant difference between 1 and 3 years in terms of change in SDMT score. The heterogeneity test *I*2 at 66.77% was considered moderate and the Egger test for publication bias was not statistically significant (*p* = .318).

## 4 DISCUSSION

This systematic review found that changes in cognitive function in MS are more often measured at the population level using a battery of tests rather than a single test. The domains most measured using a test battery were visual learning and memory, and verbal learning and memory. Many papers defined cognitive impairment as failure in 1 or more tests with a cut‐off score of < −1.5 SD from study control or normative mean and change in cognitive function as the proportion with cognitive impairment or improvement between baseline and follow‐up. This illustrates that there was no uniformity in the assessment of change in cognitive function in MS. The SDMT was the most frequently used cognitive test, either as a sole test or as part of a battery in all the papers. The meta‐analyses did not find a clinically or statistically significant change in SDMT scores over a period of three years in PwMS.

The findings suggest that there is no uniform method for measuring change in cognitive function over time. Nor is there a uniform or agreed method to denote the presence of cognitive impairment. Most papers included in this review defined cognitive impairment as a test score less than 1.5 standard deviations from a study or normative sample mean in one or more cognitive function tests, and a decline was defined by a comparison of the proportion that was impaired at baseline and follow‐up. However, cut‐off scores varied with some papers using a criterion of more than 2 or as low as 1 standard deviation. Other studies used a 10 or 20% change (decrease for impairment) in the mean score or a 3‐ to 4‐point SDMT score change between baseline and follow‐up to denote change in cognitive ability. While it can be argued that part of this inconsistency may be due to the individual specifics of the test and the cognitive domains being assessed in the study, there is a clear need for a consistent definition of what constitutes a meaningful change in cognitive function. However, this consistent definition needs to consider the heterogeneity that exists between PwMS in the profile of cognitive impairments that they experience and whether the assessment is for screening purposes or is part of a more comprehensive assessment of cognitive functioning. The lack of consistency may be problematic insofar as developing or progressive cognitive impairment may be missed or conversely over‐interpreted.

This study observed that the SDMT is popularly used in studies and that there is more emphasis in the literature on assessing information processing speed in PwMS. This may be because it has been reported to be a reliable measure (Sumowski et al., 2018). By implication, the use of reliable and sensitive tools to assess change in other domains of cognitive function has become important, particularly for the clinician who is conducting a more comprehensive assessment of cognitive function. However, while poor processing speed is often thought to underlie poor functioning in other domains of cognition (e.g., it may impair learning processes), this is not always the case (Chiaravalloti & DeLuca, 2008). Hence, one cannot infer dysfunction in everyday living based on poor processing speed alone. It may also be that specific impairments in learning and memory occur without the presence of poor processing, i.e., as would occur if there are specific lesions in the brain in areas that govern these learning and memory processes.

This study pooled and evaluated longitudinal change in SDMT scores in PwMS across studies which spanned from 1 year to 3 years. No significant change in the SDMT score was observed over three years. However, this meta‐analysis would suggest that to detect a clinically meaningful change in cognitive function using SDMT, researchers may need to aim for a longer interval than three years. This raises two important considerations. First, it may be that cognitive change in MS proceeds at such slow a rate that meaningful change is not detected over a short‐to‐moderate period. Second, it may be that the SDMT is not a sensitive marker of change in cognition. Such a slow rate of change may not be easily differentiated from cognitive decline in normal aging though older PwMS decline faster than younger PwMS (Wallach et al., 2020).

An alternative explanation for the lack of change over 3 years is that there is likely to be considerable heterogeneity in the trajectories of cognitive change over time. While some PwMS may experience no change (i.e., cognition remains stable with no excess changes compared to background normal cognitive aging), others may experience cognitive decline over time. Indeed, there is now emerging evidence that early cognitive impairments (assessed using the SDMT) are an important prognostic marker of future cognitive decline and cortical thinning (Healy et al., 2021; Pitteri et al., 2017). It is also possible that future decline may be minimized in response to changes in disease‐modifying therapies or that practice effects on the tests may exist in the published studies or due to natural fluctuations. As supported by other studies, a decline in SDMT scores among PwMS is very slow, a 1‐point change in SDMT score in 10 years (Chruzander et al., 2014) and a decline of 0.22 in SDMT score per year (Fuchs et al., 2020), although not as slow as our meta‐analysis suggest, which found that SDMT score decreased by 0.03 mean score per year.

There are several limitations of this study. First, only studies with 100 or more participants were evaluated and thus the present results may not be entirely representative of the broader population of people with MS. This was done to evaluate the use of cognitive tests and batteries in large population groups. After the full‐text screening, data were extracted by one rater though the risk of bias assessment was done by two independent raters. There was no analysis of how change occurred across groups of PwMS (i.e., according to the type of MS, measures of cognitive reserve, sex‐specific or age‐related changes or extent of baseline impairments). This is owing to this diversity not being characterized in the larger samples. Only studies in English were included due to convenience though studies with a different language could be informative. However, no article was screened out in full‐text screening based on not being written in English.

A meta‐analysis was conducted for the SDMT as it has been recommended as one of the most reliable and valid tools for measuring cognitive function in MS. A meta‐analysis was not conducted for other domains of cognitive functioning because of the high heterogeneity (in tests used) in the studies included. Change in SDMT could not be reported between RRMS and the progressive types of MS because most papers either did not define the MS group or reported a combined SDMT without differentiation.

## 5 CONCLUSION

This study highlights the slow rate of change of cognition in PwMS at the population level, the plethora of tests used for measuring cognitive change, and the need for uniformity in the measurement of change in cognitive function in PwMS both when a battery of tests or a single cognitive test is used for a comprehensive assessment or screening for change in CF. There is a clear need to establish clinically meaningful change for each cognitive test in the measurement of different cognitive domains in a battery through large clinical trials. This will have positive implications for both research and clinical practice in defining and monitoring treatment or rehabilitation outcomes. Based on these findings, we recommend the use of batteries instead of a single test in the measurement of change in cognitive function in MS and an interval of at least three years and above between baseline and follow‐up. Batteries are useful in monitoring and evaluating different cognitive function domains simultaneously. Annual measurement may help plot the trajectory of individual change, and to screen for relative cognitive impairments (upon which a more thorough evaluation with a neuropsychologist is needed), although the likelihood of possible practice effects also needs to be accounted for. Likely, clinically meaningful change in cognitive function in a population of people with MS will take more than 3 years to manifest when using SDMT, although there will be wide variations in individuals in the rate of this change. Identifying subgroups with a more rapid trajectory of change may be the best strategy to assess the effectiveness of treatments and interventions that are designed to slow, halt, or improve, cognition in PwMS. There is also a place for developing more sensitive tests that are better than SDMT. In population‐based and intervention studies, larger sample sizes, longer duration of follow‐up, and the use of cognitive batteries are recommended to allow meaningful outcomes to be reported. Based on the reviewed papers, there is no battery of tests that appears to perform better, although further work is required to determine the optimal measurement of cognitive change in this complex field.

## AUTHOR CONTRIBUTIONS

**Chigozie Ezegbe**: conceptualization; data curation; investigation; methodology; writing—original draft; writing—review & editing. **Amin Zarghami**: data curation; writing—review & editing. **Ingrid van der Mei**: methodology; writing—review & editing. **Jane Alty**: methodology; writing—review & editing. **Cynthia Honan**: conceptualization; methodology; writing—review & editing. **Bruce Taylor**: conceptualization; methodology; supervision; writing—review & editing.

## FUNDING

No funds were received for this review.

## PROSPERO

Registration CRD42021255389.

## CONFLICT OF INTEREST STATEMENT

There is no conflict of interest to declare. There was no financial support for this review.

### PEER REVIEW

The peer review history for this article is available at https://publons.com/publon/10.1002/brb3.3009.

## Supporting information

Supplementary information
