# Supplementary material for: Instruments measuring change in cognitive function in multiple sclerosis: A systematic review
Source: Brain Behav. 2023 Apr 16;13(6):e3009. doi: 10.1002/brb3.3009 (PMC10275522; doi:10.1002/brb3.3009)
Supplement: Supplementary file 1 — Supplementary information [file BRB3-13-e3009-s001.docx]

**Supplementary tables**

**Table S1 Risk of bias assessment for cohort studies/ longitudinal studies**

| Citation | Selection | | | | | | | | | | | | Comparability | | | Outcome | | | | | | | | | Total |
| --- | --- | --- | --- | --- | --- | --- | --- | --- | --- | --- | --- | --- | --- | --- | --- | --- | --- | --- | --- | --- | --- | --- | --- | --- | --- |
|  | Representativeness of the exposed cohort | | | Selection of the non-exposed cohort | | | Ascertainment of exposure | | | Demonstration that outcome of interest was not present at the start of the study | | | Based on design and analysis | | | Assessment of outcome | | | Was follow-up long enough for outcomes to occur | | | Adequacy of follow-up cohorts | | |  |
|  | Acceptable criteria | Reasoning | Point | Acceptable criteria | Reasoning | Point | Acceptable criteria | Reasoning | Point | Acceptable criteria | Reasoning | Point | Acceptable criteria | Reasoning | Point | Acceptable criteria | Reasoning | Point | Acceptable criteria | Reasoning | Point | Acceptable criteria | Reasoning | Point |  |
| Amato et al (2010) ^1^ | The study sample should be representative of the PwMS in the community | Participants included all the childhood and juvenile MS cases referred to 11 MS  Centres between January 2006 and June 2007. | * | Derived from the same population | drawn from the same community as the exposed cohort | * | Reference to primary record sources such as  medical/hospital records | Participants referred to MS Centers | * | Assessment of baseline cognitive function is necessary | Baseline assessments were compared to Follow-up assessment | * | Exposed and unexposed participants need to be matched on important factors | Exposed and unexposed participants were matched on demographic factors | * | Assessment using validated tools | Validated tools were used | ***** | Follow up period needs to be adequate for the outcome of interest to manifest | Yes, more than a 1 year | ***** | Participants lost to follow-up will need to be reported | subjects lost to follow-up unlikely to introduce bias - a small number lost (8% in the exposed group) | * | **8** |
| Amato et al (2010) ^2^ | The study sample should be representative of the PwMS in the community | A selected group of participants | - | Derived from the same population | Drawn from the same community as the exposed cohort | * | Reference to primary record sources such as  medical/hospital records | Participants referred to MS Centers | * | Assessment of baseline cognitive function is necessary | Baseline assessments were compared to Follow-up assessment | * | Exposed and unexposed participants need to be matched on important factors | Exposed and unexposed participants were matched on demographic factors | * | Assessment using validated tools | Validated tools were used | ***** | Follow up period needs to be adequate for the outcome of interest to manifest | Yes, more than a 1 year | ***** | Participants lost to follow-up will need to be reported | subjects lost to follow-up unlikely to introduce bias - a small number lost (8% in the exposed group) | * | **7** |
| Amato et al (1998) ^3^ | The study sample should be representative of the PwMS in the community | No description of the derivation of the cohort | - | Derived from the same population | No description of the derivation of the non-exposed cohort | - | Reference to primary record sources such as  medical/hospital records | No description | - | Assessment of baseline cognitive function is necessary | Baseline assessments were compared to Follow-up assessment | * | Exposed and unexposed participants need to be matched on important factors | No description | - | Assessment using validated tools | Validated tools were used | ***** | Follow up period needs to be adequate for the outcome of interest to manifest | Yes, more than a 1 year | ***** | Participants lost to follow-up will need to be reported | subjects lost to follow-up unlikely to introduce bias - small number lost (<2% in the exposed group) | * | **4** |
| Amato et al (1995) ^4^ | The study sample should be representative of the PwMS in the community | No description of the derivation of the cohort | - | Derived from the same population | Drawn from the same community as the exposed cohort | * | Reference to primary record sources such as  medical/hospital records | Validated criteria (Poser’s criteria) were used to ascertain the diagnosis of MS | * | Assessment of baseline cognitive function is necessary | Baseline assessments were compared to Follow-up assessment | * | Exposed and unexposed participants need to be matched on important factors | Exposed and unexposed participants were matched on demographic factors | * | Assessment using validated tools | Validated tools were used | ***** | Follow up period needs to be adequate for the outcome of interest to manifest | Yes, more than a 1 year | ***** | Participants lost to follow-up will need to be reported | subjects lost to follow-up unlikely to introduce bias | ***** | **7** |
| Amato et al (2001) ^5^ | The study sample should be representative of the PwMS in the community | No description of the derivation of the cohort | - | Derived from the same population | Drawn from the same community as the exposed cohort | * | Reference to primary record sources such as  medical/hospital records | No description | - | Assessment of baseline cognitive function is necessary | Baseline assessments were compared to Follow-up assessment | * | Exposed and unexposed participants need to be matched on important factors | Exposed and unexposed participants were matched on demographic factors | * | Assessment using validated tools | Validated tools were used | ***** | Follow up period needs to be adequate for the outcome of interest to manifest | Yes, more than a 1 year | ***** | Participants lost to follow-up will need to be reported | subjects lost to follow-up unlikely to introduce bias (<10%) | ***** | **5** |
| Beckerman et al. (2013) ^6^ | The study sample should be representative of the PwMS in the community | A selected group of participants | - | Derived from the same population | No description of the derivation of the non-exposed cohort | - | Secured records, Structured interviews, properly diagnosed | Had a definite diagnosis of MS. MS type was further determined by a neurologist using a standardised definition | * | Assessment of baseline cognitive function is necessary | Baseline assessments were compared to Follow-up assessment | * | Exposed and unexposed participants need to be matched on important factors | NA | - | Assessment using validated tools | Validated tools were used | ***** | Follow up period needs to be adequate for the outcome of interest to manifest | Yes, more than a 1 year | ***** | Participants lost to follow-up will need to be reported | subjects lost to follow-up unlikely to introduce bias - small number lost –(10% of total measurements) description provided of those lost) | ***** | **5** |
| Borghi et al. (2016) ^7^ | The study sample should be representative of the PwMS in the community | Convenience sample (somewhat representative of the PwMS in the community) | ***** | Derived from the same population | drawn from the same community as the exposed cohort | * | Secured records, Structured interviews, properly diagnosed | patients underwent detailed biological and clinical investigations,  and received a diagnosis of MS | ***** | Assessment of baseline cognitive function is necessary | Baseline assessments were compared to Follow-up assessment | * | Exposed and unexposed participants need to be matched on important factors | Exposed and unexposed participants were matched on demographic factors | * | Assessment using validated tools | Validated tools were used | ***** | Follow up period needs to be adequate for the outcome of interest to manifest | Yes, more than a 1 year | ***** | Participants lost to follow-up will need to be reported | Subjects lost to follow up likely to introduce bias (28%) | **-** | **6** |
| Bosma et al. (2010) ^8^ | The study sample should be representative of the PwMS in the community | A selected group of participants | **-** | Derived from the same population | No description of the derivation of the non-exposed cohort | - | Secured records, Structured interviews, properly diagnosed | Had a definite diagnosis of MS | ***** | Assessment of baseline cognitive function is necessary | Baseline assessments were compared to Follow-up assessment | ***** | Exposed and unexposed participants need to be matched on important factors | NA | **-** | Assessment using validated tools | Validated tools were used | ***** | Follow up period needs to be adequate for the outcome of interest to manifest | Yes, more than a 1 year | ***** | Participants lost to follow-up will need to be reported | This is a retrospective cohort study (loss to follow-up was not assessed and reported) | **-** | **4** |
| Bsteh et al. (2019) ^9^ | The study sample should be representative of the PwMS in the community | A selected group of participants | **-** | Derived from the same population | No description of the derivation of the non-exposed cohort | - | Secured records, Structured interviews, properly diagnosed | Had a definite diagnosis of MS according to the 2010 McDonald criteria | ***** | Assessment of baseline cognitive function is necessary | Baseline assessments were compared to Follow-up assessment | ***** | Exposed and unexposed participants need to be matched on important factors | NA | **-** | Assessment using validated tools | Validated tools were used | ***** | Follow up period needs to be adequate for the outcome of interest to manifest | Yes, more than a 1 year | ***** | Participants lost to follow-up will need to be reported | Subjects lost to follow up unlikely to introduce bias (<10%) | ***** | **5** |
| Chruzander et al. (2014) ^10^ | The study sample should be representative of the PwMS in the community | A random sampling of a large population | ***** | Derived from the same population | No description of the derivation of the non-exposed cohort | - | Secured records, Structured interviews, properly diagnosed | Had a definite diagnosis of MS | ***** | Assessment of baseline cognitive function is necessary | Baseline assessments were compared to Follow-up assessment | ***** | Exposed and unexposed participants need to be matched on important factors | NA | **-** | Assessment using validated tools | Validated tools were used | ***** | Follow up period needs to be adequate for the outcome of interest to manifest | Yes, more than a 1 year | ***** | Participants lost to follow-up will need to be reported | Follow-up was complete and missing data reported | ***** | **6** |
| Crielaard et al. (2019) ^11^ | The study sample should be representative of the PwMS in the community | Large population sample MS registry | ***** | Derived from the same population | No description of the derivation of the non-exposed cohort | - | Secured records, Structured interviews, properly diagnosed | Had a definite diagnosis of MS | ***** | Assessment of baseline cognitive function is necessary | Baseline assessments were compared to Follow-up assessment | ***** | Exposed and unexposed participants need to be matched on important factors | NA | **-** | Assessment using validated tools | Validated tools were used | ***** | Follow up period needs to be adequate for the outcome of interest to manifest | Yes, more than a 1 year | ***** | Participants lost to follow-up will need to be reported | This is a retrospective cohort study (loss to follow-up was not assessed and reported) | **-** | **5** |
| de Groot et al. (2009) ^12^ | The study sample should be representative of the PwMS in the community | A selected group of participants | **-** | Derived from the same population | No description of the derivation of the non-exposed cohort | - | Secured records, Structured interviews, properly diagnosed | Had a definite diagnosis of MS according to the Poser criteria | ***** | Assessment of baseline cognitive function is necessary | Baseline assessments were compared to Follow-up assessment | ***** | Exposed and unexposed participants need to be matched on important factors | NA | **-** | Assessment using validated tools | Validated tools were used | ***** | Follow up period needs to be adequate for the outcome of interest to manifest | Yes, more than a 1 year | ***** | Participants lost to follow-up will need to be reported | “To improve data quality and reduce the risk of bias,  missing data on predictors were imputed” | ***** | **5** |
| De Meijer et al. (2018) ^13^ | The study sample should be representative of the PwMS in the community | A selected group of participants | **-** | Derived from the same population | drawn from the same community as the exposed cohort | * | Secured records, Structured interviews, properly diagnosed | Participants were chosen from a Tertiary Hospital clinic | * | Assessment of baseline cognitive function is necessary | Baseline assessments were compared to Follow-up assessment | ***** | Exposed and unexposed participants need to be matched on important factors | Exposed and unexposed participants were matched on demographic factors | * | Assessment using validated tools | Validated tools were used | ***** | Follow up period needs to be adequate for the outcome of interest to manifest | Yes, (1 year) | ***** | Participants lost to follow-up will need to be reported | Follow-up was complete | ***** | **7** |
| Demakis et al. (2010) ^14^ | The study sample should be representative of the PwMS in the community | Convenience sample (large sample size, somewhat representative of the average in the community) | ***** | Derived from the same population | drawn from the same community as the exposed cohort | * | Secured records, Structured interviews, properly diagnosed | Had a definite diagnosis of MS | ***** | Assessment of baseline cognitive function is necessary | Baseline assessments were compared to Follow-up assessment | ***** | Exposed and unexposed participants need to be matched on important factors | the study controlled for age and education | ***** | Assessment using validated tools | Validated tools were used | ***** | Follow up period needs to be adequate for the outcome of interest to manifest | Yes, more than a 1 year | ***** | Participants lost to follow-up will need to be reported | no statement | **-** | **7** |
| Demakis et al. (2009) ^15^ | The study sample should be representative of the PwMS in the community | Convenience sample (large sample size, somewhat representative of the average in the community) | ***** | Derived from the same population | No description of the derivation of the non-exposed cohort | - | Secured records, Structured interviews, properly diagnosed | Had a definite diagnosis of MS | ***** | Assessment of baseline cognitive function is necessary | Baseline assessments were compared to Follow-up assessment | ***** | Exposed and unexposed participants need to be matched on important factors | NA | **-** | Assessment using validated tools | Validated tools were used | ***** | Follow up period needs to be adequate for the outcome of interest to manifest | Yes, more than a 1 year | ***** | Participants lost to follow-up will need to be reported | Follow-up was complete and missing data reported | **-** | **5** |
| Eijlers et al. (2018) ^16^ | The study sample should be representative of the PwMS in the community | No description of the derivation of the cohort | - | Derived from the same population | drawn from the same community as the exposed cohort | * | Reference to primary record sources such as  medical/hospital records | No description | - | Assessment of baseline cognitive function is necessary | Baseline assessments were compared to Follow-up assessment | * | Exposed and unexposed participants need to be matched on important factors | No description | **-** | Assessment using validated tools | Validated tools were used | ***** | Follow up period needs to be adequate for the outcome of interest to manifest | Yes, more than a 1 year | ***** | Participants lost to follow-up will need to be reported | Complete follow-up - all subjects accounted for | ***** | **5** |
| Fuchs et al. (2020) ^17^ | The study sample should be representative of the PwMS in the community | A selected group of participants | **-** | Derived from the same population | No description of the derivation of the non-exposed cohort | - | Secured records, Structured interviews, properly diagnosed | Had a definite diagnosis of MS | ***** | Assessment of baseline cognitive function is necessary | Baseline assessments were compared to Follow-up assessment | ***** | Exposed and unexposed participants need to be matched on important factors | NA | **-** | Assessment using validated tools | Validated tools were used | ***** | Follow up period needs to be adequate for the outcome of interest to manifest | Yes, more than a 1 year | ***** | Participants lost to follow-up will need to be reported | Follow-up was complete and missing data reported | ***** | **5** |
| Healy et al. (2021) ^18^ | The study sample should be representative of the PwMS in the community | A selected group of participants | **-** | Derived from the same population | No description of the derivation of the non-exposed cohort | - | Secured records, Structured interviews, properly diagnosed | Had a definite diagnosis of MS | ***** | Assessment of baseline cognitive function is necessary | Baseline assessments were compared to Follow-up assessment | ***** | Exposed and unexposed participants need to be matched on important factors | NA | **-** | Assessment using validated tools | Validated tools were used | ***** | Follow up period needs to be adequate for the outcome of interest to manifest | More than a 1 year | ***** | Participants lost to follow-up will need to be reported | No statement | **-** | **4** |
| Heled et al. (2021) ^19^ | The study sample should be representative of the PwMS in the community | A selected group of participants | **-** | Derived from the same population | No description of the derivation of the non-exposed cohort | - | Secured records, Structured interviews, properly diagnosed | Had a definite diagnosis of MS | ***** | Assessment of baseline cognitive function is necessary | Baseline assessments of cognition were performed and reported | ***** | Exposed and unexposed participants need to be matched on important factors | NA | **-** | Assessment using validated tools | Validated tools were used | ***** | Follow up period needs to be adequate for the outcome of interest to manifest | More than a 1 year | ***** | Participants lost to follow-up will need to be reported | Follow-up was complete and missing data reported | ***** | **5** |
| Hoogervorst et al. (2002) ^20^ | The study sample should be representative of the PwMS in the community | A selected group of participants | **-** | Derived from the same population | No description of the derivation of the non-exposed cohort | - | Secured records, Structured interviews, properly diagnosed | Had a definite diagnosis of MS | ***** | Assessment of baseline cognitive function is necessary | Baseline assessments of cognition were performed and reported | ***** | Exposed and unexposed participants need to be matched on important factors | NA | **-** | Assessment using validated tools | Validated tools were used | ***** | Follow up period needs to be adequate for the outcome of interest to manifest | More than a 1 year | ***** | Participants lost to follow-up will need to be reported | Subjects lost to follow up unlikely to introduce bias (<10%) | ***** | **5** |
| Hughes et al. (2015) ^21^ | The study sample should be representative of the PwMS in the community | A selected group of participants | **-** | Derived from the same population | No description of the derivation of the non-exposed cohort | - | Secured records, Structured interviews, properly diagnosed | Had a definite diagnosis of MS | ***** | Assessment of baseline cognitive function is necessary | Baseline assessments were done and reported | ***** | Exposed and unexposed participants need to be matched on important factors | NA | **-** | Assessment using validated tools | Validated tools were used | ***** | Follow up period needs to be adequate for the outcome of interest to manifest | More than a 1 year | ***** | Participants lost to follow-up will need to be reported | Subjects lost to follow up likely to introduce bias | **-** | **4** |
| Hughes et al. (2018) ^22^ | The study sample should be representative of the PwMS in the community | A selected group of participants | **-** | Derived from the same population | No description of the derivation of the non-exposed cohort | - | Secured records, Structured interviews, properly diagnosed | Had a definite diagnosis of MS | ***** | Assessment of baseline cognitive function is necessary | Baseline assessments were done and reported | ***** | Exposed and unexposed participants need to be matched on important factors | NA | **-** | Assessment using validated tools | Validated tools were used | ***** | Follow up period needs to be adequate for the outcome of interest to manifest | More than a 1 year | ***** | Participants lost to follow-up will need to be reported | Subjects lost to follow up unlikely to introduce bias (<10%) | ***** | **5** |
| Jakimovski et al. (2020) ^23^ | The study sample should be representative of the PwMS in the community | No description of the derivation of the cohort | - | Derived from the same population | No description of the derivation of the non-exposed cohort | - | Reference to primary record sources such as  medical/hospital records | No description | - | Assessment of baseline cognitive function is necessary | Baseline assessments were compared to Follow-up assessment | * | Exposed and unexposed participants need to be matched on important factors | the study controlled for age and education | ***** | Assessment using validated tools | Validated tools were used | ***** | Follow up period needs to be adequate for the outcome of interest to manifest | Yes, more than a 1 year | ***** | Participants lost to follow-up will need to be reported | Complete follow-up - all subjects accounted for | ***** | **5** |
| Johansson et al. (2020) ^24^ | The study sample should be representative of the PwMS in the community | A selected group of participants | **-** | Derived from the same population | No description of the derivation of the non-exposed cohort | - | Secured records, Structured interviews, properly diagnosed | Had a definite diagnosis of MS | ***** | Assessment of baseline cognitive function is necessary | Baseline assessments were done and reported | ***** | Exposed and unexposed participants need to be matched on important factors | NA | **-** | Assessment using validated tools | Validated tools were used | ***** | Follow up period needs to be adequate for the outcome of interest to manifest | More than a 1 year | ***** | Participants lost to follow-up will need to be reported | Subjects lost to follow up unlikely to introduce bias (<10%) |  | **4** |
| Johnen et al. (2019) ^25^ | The study sample should be representative of the PwMS in the community | (large sample size, somewhat representative of the average in the community) | ***** | Derived from the same population | No description of the derivation of the non-exposed cohort | - | Secured records, Structured interviews, properly diagnosed | Had a definite diagnosis of MS | ***** | Assessment of baseline cognitive function is necessary | Baseline assessments were done and reported | ***** | Exposed and unexposed participants need to be matched on important factors | NA | **-** | Assessment using validated tools | Validated tools were used | ***** | Follow up period needs to be adequate for the outcome of interest to manifest | More than a 1 year | ***** | Participants lost to follow-up will need to be reported | Follow-up was complete and Subjects lost to follow up unlikely to introduce bias (<10%) | ***** | **6** |
| Jønsson et al. (2006) ^26^ | The study sample should be representative of the PwMS in the community | No description of the derivation of the cohort | - | Derived from the same population | No description of the derivation of the non-exposed cohort | - | Secured records, Structured interviews, properly diagnosed | Had a definite diagnosis of MS | ***** | Assessment of baseline cognitive function is necessary | Baseline assessments were compared to Follow-up assessment | * | Exposed and unexposed participants need to be matched on important factors | No description | **-** | Assessment using validated tools | Validated tools were used | ***** | Follow up period needs to be adequate for the outcome of interest to manifest | Yes, more than a 1 year | ***** | Participants lost to follow-up will need to be reported | Subjects lost to follow up likely to introduce bias | **-** | **4** |
| López-Góngora et al. (2015) ^27^ | The study sample should be representative of the PwMS in the community | No description of the derivation of the cohort | - | Derived from the same population | Drawn from the same community as the exposed cohort | * | Secured records, Structured interviews, properly diagnosed | Had a definite diagnosis of MS | ***** | Assessment of baseline cognitive function is necessary | Baseline assessments were compared to Follow-up assessment | * | Exposed and unexposed participants need to be matched on important factors | No description | **-** | Assessment using validated tools | Validated tools were used | ***** | Follow up period needs to be adequate for the outcome of interest to manifest | Yes, more than a 1 year | ***** | Participants lost to follow-up will need to be reported | Subjects lost to follow up likely to introduce bias | **-** | **5** |
| McKay et al. (2019) ^28^ | The study sample should be representative of the PwMS in the community | Sample was representative | ***** | Derived from the same population | No description of the derivation of the non-exposed cohort | - | Secured records, Structured interviews, properly diagnosed | Had a definite diagnosis of MS | ***** | Assessment of baseline cognitive function is necessary | Baseline assessments were done and reported | ***** | Exposed and unexposed participants need to be matched on important factors | NA | **-** | Assessment using validated tools | Validated tools were used | ***** | Follow up period needs to be adequate for the outcome of interest to manifest | More than a 1 year | ***** | Participants lost to follow-up will need to be reported | Follow-up was complete and Subjects lost to follow up unlikely to introduce bias (<10%) | ***** | **6** |
| Motyl et al. (2021) ^29^ | The study sample should be representative of the PwMS in the community | (large sample size, somewhat representative of the average in the community) | ***** | Derived from the same population | No description of the derivation of the non-exposed cohort | - | Secured records, Structured interviews, properly diagnosed | Had a definite diagnosis of MS | ***** | Assessment of baseline cognitive function is necessary | Baseline assessments were done and reported | ***** | Exposed and unexposed participants need to be matched on important factors | NA | **-** | Assessment using validated tools | Validated tools were used | ***** | Follow up period needs to be adequate for the outcome of interest to manifest | More than a 1 year | ***** | Participants lost to follow-up will need to be reported | Subjects lost to follow up unlikely to introduce bias (<10%) | ***** | **6** |
| Raimo et al. (2020) ^30^ | The study sample should be representative of the PwMS in the community | A selected group of participants | **-** | Derived from the same population | No description of the derivation of the non-exposed cohort | - | Secured records, Structured interviews, properly diagnosed | Had a definite diagnosis of MS | ***** | Assessment of baseline cognitive function is necessary | Baseline assessments were done and reported | ***** | Exposed and unexposed participants need to be matched on important factors | NA | **-** | Assessment using validated tools | Validated tools were used | ***** | Follow up period needs to be adequate for the outcome of interest to manifest | More than a 1 year | ***** | Participants lost to follow-up will need to be reported | Subjects lost to follow up likely to introduce bias (>10%) | **-** | **4** |
| Roy et al. (2018) ^31^ | The study sample should be representative of the PwMS in the community | A selected group of participants | **-** | Derived from the same population | No description of the derivation of the non-exposed cohort | - | Secured records, Structured interviews, properly diagnosed | Had a definite diagnosis of MS | ***** | Assessment of baseline cognitive function is necessary | Baseline assessments were done and reported | ***** | Exposed and unexposed participants need to be matched on important factors | NA | **-** | Assessment using validated tools | Validated tools were used | ***** | Follow up period needs to be adequate for the outcome of interest to manifest | More than a 1 year | ***** | Participants lost to follow-up will need to be reported | Follow-up was complete but missing data was not reported | **-** | **4** |
| Strober et al. (2019) ^32^ | The study sample should be representative of the PwMS in the community | (Large sample size, somewhat representative of the average in the community) | ***** | Derived from the same population | No description of the derivation of the non-exposed cohort | - | Secured records, Structured interviews, properly diagnosed | Had a definite diagnosis of MS | ***** | Assessment of baseline cognitive function is necessary | Baseline assessments were done and reported | ***** | Exposed and unexposed participants need to be matched on important factors | NA | **-** | Assessment using validated tools | Validated tools were used | ***** | Follow up period needs to be adequate for the outcome of interest to manifest | More than a 1 year | ***** | Participants lost to follow-up will need to be reported | Follow-up was complete but missing data was not reported | **-** | **5** |
| Uher et al. (2017) ^33^ | The study sample should be representative of the PwMS in the community | (Large sample size, somewhat representative of the average in the community) | ***** | Derived from the same population | No description of the derivation of the non-exposed cohort | - | Secured records, Structured interviews, properly diagnosed | Had a definite diagnosis of MS | ***** | Assessment of baseline cognitive function is necessary | Baseline assessments were done and reported | ***** | Exposed and unexposed participants need to be matched on important factors | NA | **-** | Assessment using validated tools | Validated tools were used | ***** | Follow up period needs to be adequate for the outcome of interest to manifest | More than a 1 year | ***** | Participants lost to follow-up will need to be reported | Follow-up was complete and missing data reported | ***** | **6** |
| Wallach et al. (2020) ^34^ | The study sample should be representative of the PwMS in the community | Somewhat representative of the average paediatric onset MS in the community | ***** | Derived from the same population | No description of the derivation of the non-exposed cohort | - | Secured records, Structured interviews, properly diagnosed | Had a definite diagnosis of MS | ***** | Assessment of baseline cognitive function is necessary | Baseline assessments were done and reported | ***** | Exposed and unexposed participants need to be matched on important factors | NA | **-** | Assessment using validated tools | Validated tools were used | ***** | Follow up period needs to be adequate for the outcome of interest to manifest | More than a 1 year | ***** | Participants lost to follow-up will need to be reported | Follow-up was complete and the two groups did not differ with  respect to the main contributing factors | ***** | **6** |
| Wu et al (2020) ^35^ | The study sample should be representative of the PwMS in the community | A selected group of participants | **-** | Derived from the same population | No description of the derivation of the non-exposed cohort | - | Secured records, Structured interviews, properly diagnosed | self-reporting a confirmed diagnosis of MS. | **-** | Assessment of baseline cognitive function is necessary | Baseline assessments were done and reported | ***** | Exposed and unexposed participants need to be matched on important factors | NA | **-** | Assessment using validated tools | Validated tools were used | ***** | Follow up period needs to be adequate for the outcome of interest to manifest | More than a 1 year | ***** | Participants lost to follow-up will need to be reported | Follow-up was complete and missing data reported | ***** | **4** |
| Ytterberg et al. (2008) ^36^ | The study sample should be representative of the PwMS in the community | A selected group of participants | **-** | Derived from the same population | No description of the derivation of the non-exposed cohort | - | Secured records, Structured interviews, properly diagnosed | Had a definite diagnosis of MS | ***** | Assessment of baseline cognitive function is necessary | Baseline assessments were done and reported | ***** | Exposed and unexposed participants need to be matched on important factors | NA | **-** | Assessment using validated tools | Validated tools were used | ***** | Follow up period needs to be adequate for the outcome of interest to manifest | More than a 1 year | ***** | Participants lost to follow-up will need to be reported | Follow-up was complete and subjects lost to follow up unlikely to introduce bias (<10%) | ***** | **5** |
| Benešová et al. (2017) ^37^ | The study sample should be representative of the PwMS in the community | A selected group of participants | **-** | Derived from the same population | No description of the derivation of the non-exposed cohort | - | Secured records, Structured interviews, properly diagnosed | Fulfilled McDonald’s criteria for MS | ***** | Assessment of baseline cognitive function is necessary | Baseline assessments were done and reported | ***** | Exposed and unexposed participants need to be matched on important factors | NA | **-** | Assessment using validated tools | Validated tools were used | ***** | Follow up period needs to be adequate for the outcome of interest to manifest | More than a 1 year | ***** | Participants lost to follow-up will need to be reported | Follow-up was complete and subjects lost to follow up unlikely to introduce bias (<10%) | ***** | **5** |
| Iaffaldano et al. (2012) ^38^ | The study sample should be representative of the PwMS in the community | A selected group of participants | **-** | Derived from the same population | No description of the derivation of the non-exposed cohort | - | Secured records, Structured interviews, properly diagnosed | Had a definite diagnosis of MS | ***** | Assessment of baseline cognitive function is necessary | Baseline assessments were done and reported | ***** | Exposed and unexposed participants need to be matched on important factors | NA | **-** | Assessment using validated tools | Validated tools were used | ***** | Follow up period needs to be adequate for the outcome of interest to manifest | More than a 1 year | ***** | Participants lost to follow-up will need to be reported | Participants that completed the follow-up | ***** | **5** |
| Koch et al. (2021) ^39^ | The study sample should be representative of the PwMS in the community | A selected group of participants | **-** | Derived from the same population | No description of the derivation of the non-exposed cohort | - | Secured records, Structured interviews, properly diagnosed | Had a definite diagnosis of MS | ***** | Assessment of baseline cognitive function is necessary | Baseline assessments were done and reported | ***** | Exposed and unexposed participants need to be matched on important factors | NA | **-** | Assessment using validated tools | Validated tools were used | ***** | Follow up period needs to be adequate for the outcome of interest to manifest | More than a 1 year | ***** | Participants lost to follow-up will need to be reported | Subjects lost to follow up likely to introduce bias (>10%) |  | **4** |
| Patti et al. (2013) ^40^ | The study sample should be representative of the PwMS in the community | A selected group of participants | **-** | Derived from the same population | No description of the derivation of the non-exposed cohort | - | Secured records, Structured interviews, properly diagnosed | Fulfilled McDonald’s criteria for MS | ***** | Assessment of baseline cognitive function is necessary | Baseline assessments were done and reported | ***** | Exposed and unexposed participants need to be matched on important factors | NA | **-** | Assessment using validated tools | Validated tools were used | ***** | Follow up period needs to be adequate for the outcome of interest to manifest | More than a 1 year | ***** | Participants lost to follow-up will need to be reported | Subjects lost to follow up likely to introduce bias (>10%) | **-** | **4** |
| Perumal et al. (2019) ^41^ | The study sample should be representative of the PwMS in the community | A selected group of participants | **-** | Derived from the same population | No description of the derivation of the non-exposed cohort | - | Secured records, Structured interviews, properly diagnosed | Had a definite diagnosis of MS | ***** | Assessment of baseline cognitive function is necessary | Baseline assessments were done and reported | ***** | Exposed and unexposed participants need to be matched on important factors | NA | **-** | Assessment using validated tools | Validated tools were used | ***** | Follow up period needs to be adequate for the outcome of interest to manifest | More than a 1 year | ***** | Participants lost to follow-up will need to be reported | Subjects lost to follow up likely to introduce bias (>10%) | **-** | **4** |
| Rudick et al. (2009) ^42^ | The study sample should be representative of the PwMS in the community | A selected group of participants | **-** | Derived from the same population | No description of the derivation of the non-exposed cohort | - | Secured records, Structured interviews, properly diagnosed | Had a definite diagnosis of MS | ***** | Assessment of baseline cognitive function is necessary | Baseline assessments were done and reported | ***** | Exposed and unexposed participants need to be matched on important factors | NA | **-** | Assessment using validated tools | Validated tools were used | ***** | Follow up period needs to be adequate for the outcome of interest to manifest | More than a 1 year | ***** | Participants lost to follow-up will need to be reported | No statement | **-** | **4** |
| Schwartz et al. (1996) ^43^ | The study sample should be representative of the PwMS in the community | A selected group of participants | ***** | Derived from the same population | No description of the derivation of the non-exposed cohort | - | Secured records, Structured interviews, properly diagnosed | Had a definite diagnosis of MS | ***** | Assessment of baseline cognitive function is necessary | Baseline assessments were done and reported | ***** | Exposed and unexposed participants need to be matched on important factors | NA | **-** | Assessment using validated tools | Validated tools were used | ***** | Follow up period needs to be adequate for the outcome of interest to manifest | More than a 1 year | ***** | Participants lost to follow-up will need to be reported | No statement | **-** | **5** |
| Stephenson et al. (2012) ^44^ | The study sample should be representative of the PwMS in the community | A selected group of participants | **-** | Derived from the same population | No description of the derivation of the non-exposed cohort | - | Secured records, Structured interviews, properly diagnosed | Had a definite diagnosis of MS | ***** | Assessment of baseline cognitive function is necessary | Baseline assessments were done and reported | ***** | Exposed and unexposed participants need to be matched on important factors | NA | **-** | Assessment using validated tools | Validated tools were used | ***** | Follow up period needs to be adequate for the outcome of interest to manifest | More than a 1 year | ***** | Participants lost to follow-up will need to be reported | Subjects lost to follow up likely to introduce bias (>10%) | **-** | **4** |

Table S2. Risk of bias assessment for Randomized Controlled Trial publications

| Citation (first author) | Selection | | | | | | | | | | | | Comparability | | | Exposure | | | | | | Total |
| --- | --- | --- | --- | --- | --- | --- | --- | --- | --- | --- | --- | --- | --- | --- | --- | --- | --- | --- | --- | --- | --- | --- |
|  | Is the Case Definition Adequate? | | | Representativeness of the Cases | | | Selection of Controls | | | Definition of Controls | | | Comparability of Cases and Controls based on the design or analysis | | | Ascertainment of Exposure/ Same method of ascertainment for cases and controls | | | Non-Response Rate | | |  |
|  | Acceptable criteria | Reasoning | Point | Acceptable criteria | Reasoning | Point | Acceptable criteria | Reasoning | Point | Acceptable criteria | Reasoning | Point | Acceptable criteria | Reasoning | Point | Acceptable criteria | Reasoning | Point | Acceptable criteria | Reasoning | Point |  |
| Benedict et al. (2021) ^45^ | Reference to primary record sources such as  medical/hospital records | Yes, with validation | * | All eligible cases or an appropriate sample of those  cases (e.g. random sample) | No information provided | **-** | Community controls | Same MS patients randomized 2:1 | * | Must make clear that controls  have no history of this outcome | NA | **-** | Cases and controls must be matched in the design and/or confounders must be  adjusted for in the analysis | No information provided | - | Secure record, Structured interview by  healthcare practitioner, blind to  case/control status (Same for both groups) | Validated tools were used | ** | Same for both groups | Similar for each group | ***** | **5** |
| Benedict et al. (2018) ^46^ | Reference to primary record sources such as  medical/hospital records | Yes, with validation | * | All eligible cases or an appropriate sample of those  cases (e.g. random sample) | Random sample | ***** | Community controls | Same MS patients randomized 1:1 | * | Must make clear that controls  have no history of this outcome | NA | **-** | Cases and controls must be matched in the design and/or confounders must be  adjusted for in the analysis | Demographics were well balanced | * | Secure record, Structured interview by  healthcare practitioner, blind to  case/control status (Same for both groups) | Validated tools were used | ** | Same for both groups | No information provided | **-** | **6** |
| Chan et al. (2017) ^47^ | Reference to primary record sources such as  medical/hospital records | Fulfilled the revised McDonald diagnostic criteria | * | All eligible cases or an appropriate sample of those  cases (e.g. random sample) | Random sample | ***** | Community controls | Same MS patients randomized 1:1 | * | Must make clear that controls  have no history of this outcome | NA | **-** | Cases and controls must be matched in the design and/or confounders must be  adjusted for in the analysis | No information provided | - | Secure record, Structured interview by  healthcare practitioner, blind to  case/control status (Same for both groups) | Validated tools were used | ** | Same for both groups | Similar for each group | ***** | **6** |
| Cinar et al. (2017) ^48^ | Reference to primary record sources such as  medical/hospital records | Fulfilled McDonald’s criteria for MS | * | All eligible cases or an appropriate sample of those  cases (e.g. random sample) | Convenience sample | - | Community controls | Participant relatives and hospital employees | ***** | Must explicitly state that controls  have no history of this outcome | Controls stated to be healthy | ***** | Cases and controls must be matched in the design and/or confounders must be  adjusted for in the analysis | Cases and controls were matched on age ‘sex and education | * | Secure record, Structured interview by  healthcare practitioner, blind to  case/control status (Same for both groups) | Validated tools were used | ** | Same for both groups | The difference in attrition between the MS group and the control group was not mentioned | **-** | **6** |
| Comi et al. (2017) ^49^ | Reference to primary record sources such as  medical/hospital records | Fulfilled McDonald’s criteria for MS | ***** | All eligible cases or an appropriate sample of those  cases (e.g. random sample) | Convenience sample | **-** | Community controls | Same MS patients randomized 2:1 | * | Must make clear that controls  have no history of this outcome | NA | **-** | Cases and controls must be matched in the design and/or confounders must be | No information provided | - | Secure record, Structured interview by  healthcare practitioner, blind to  case/control status (Same for both groups) | Validated tools were used | ** | Same for both groups | Higher for one group compared to the other (8.49% vs 41.18%) | **-** | **4** |
| DeLuca et al. (2021) ^50^ | Reference to primary record sources such as  medical/hospital records | Fulfilled the revised McDonald diagnostic criteria | * | All eligible cases or an appropriate sample of those  cases (e.g. random sample) | Random sample | ***** | Community controls | Same MS patients randomized 1:1:1 | * | Must make clear that controls  have no history of this outcome | NA | **-** | Cases and controls must be matched in the design and/or confounders must be  adjusted for in the analysis | No information provided | - | Secure record, Structured interview by  healthcare practitioner, blind to  case/control status (Same for both groups) | Validated tools were used | ** | Same for both groups | No information provided | **-** | **5** |
|  |  |  |  |  |  |  |  |  |  |  |  |  |  |  |  |  |  |  |  |  |  |  |
| De Giglio et al. (2016) ^51^ | Reference to primary record sources such as  medical/hospital records | Fulfilled the revised McDonald diagnostic criteria | * | All eligible cases or an appropriate sample of those  cases (e.g. random sample) | Convenience sample | **-** | Community controls | Same MS patients randomized 1:1:1 | * | Must make clear that controls  have no history of this outcome | NA | **-** | Cases and controls must be matched in the design and/or confounders must be  adjusted for in the analysis | Adjusted for study group, age, years in formal education and number of the impaired test at baseline | * | Secure record, Structured interview by  healthcare practitioner, blind to  case/control status (Same for both groups) | Validated tools were used | ** | Same for both groups | Similar for both groups | ***** | **6** |
| Fischer et al. (2000) ^52^ | Reference to primary record sources such as  medical/hospital records | Documented evidence of MS | * | All eligible cases or an appropriate sample of those  cases (e.g. random sample) | Convenience sample | **-** | Community controls | Same MS patients randomized | * | Must make clear that controls  have no history of this outcome | NA | **-** | Cases and controls must be matched in the design and/or confounders must be  adjusted for in the analysis | Adjusted for age, education and gender | * | Secure record, Structured interview by  healthcare practitioner, blind to  case/control status (Same for both groups) | Validated tools were used | ** | Same for both groups | No information provided | ***** | **5** |
| Lincoln et al. (2020) ^53^ | Reference to primary record sources such as  medical/hospital records | Documented evidence of MS | * | All eligible cases or an appropriate sample of those  cases (e.g. random sample) | Convenience sample | **-** | Community controls | Same MS patients randomized 6:5 | * | Must make clear that controls  have no history of this outcome | NA | **-** | Cases and controls must be matched in the design and/or confounders must be  adjusted for in the analysis | Adjusted for age, education and gender | * | Secure record, Structured interview by  healthcare practitioner, blind to  case/control status (Same for both groups) | Validated tools were used | ** | Same for both groups | No difference between the groups | ***** | **6** |
| Patti et al. (2010) ^54^ | Reference to primary record sources such as  medical/hospital records | Fulfilled the McDonald's diagnostic criteria | * | All eligible cases or an appropriate sample of those  cases (e.g. random sample) | Convenience sample | **-** | Community controls | Same MS patients randomized | * | Must make clear that controls  have no history of this outcome | NA | **-** | Cases and controls must be matched in the design and/or confounders must be  adjusted for in the analysis | Adjusted for confounders | * | Secure record, Structured interview by  healthcare practitioner, blind to  case/control status (Same for both groups) | Validated tools were used | ** | Same for both groups | Dropout was similar in both groups | ***** | **6** |
| Schwid et al. (2007) ^55^ | Reference to primary record sources such as  medical/hospital records | Definite diagnosis of MS | * | All eligible cases or an appropriate sample of those  cases (e.g. random sample) | Convenience sample | **-** | Community controls | Same MS patients randomized | * | Must make clear that controls  have no history of this outcome | NA | **-** | Cases and controls must be matched in the design and/or confounders must be  adjusted for in the analysis | Adjusted for confounders | * | Secure record, Structured interview by  healthcare practitioner, blind to  case/control status (Same for both groups) | Validated tools were used | ** | Same for both groups | No information provided | **-** | **5** |
| Weinstein et al. (1999) ^56^ | Reference to primary record sources such as  medical/hospital records | clinically definite MS | * | All eligible cases or an appropriate sample of those  cases (e.g. random sample) | Convenience sample | **-** | Community controls | Same MS patients randomized | * | Must make clear that controls  have no history of this outcome | NA | **-** | Cases and controls must be matched in the design and/or confounders must be  adjusted for in the analysis | No information provided | - | Secure record, Structured interview by  healthcare practitioner, blind to  case/control status (Same for both groups) | Validated tools were used | ** | Same for both groups | No information provided | **-** | **4** |
| Weinstock-Guttman et al (2012) ^57^ | Reference to primary record sources such as  medical/hospital records | Fulfilled the McDonald's diagnostic criteria | * | All eligible cases or an appropriate sample of those  cases (e.g. random sample) | Multicenter sample | ***** | Community controls | Same MS patients randomized | * | Must make clear that controls  have no history of this outcome | NA | **-** | Cases and controls must be matched in the design and/or confounders must be  adjusted for in the analysis | No information provided | - | Secure record, Structured interview by  healthcare practitioner, blind to  case/control status (Same for both groups) | Validated tools were used | ** | Same for both groups | No information provided | **-** | **5** |

**References**

**1.** Amato MP, Goretti B, Ghezzi A, et al. Cognitive and psychosocial features in childhood and juvenile MS: two-year follow-up. *Neurology.* 2010;75(13):1134-1140.

**2.** Amato MP, Portaccio E, Goretti B, et al. Relevance of cognitive deterioration in early relapsing-remitting MS: a 3-year follow-up study. *Mult Scler.* 2010;16(12):1474-1482.

**3.** Amato MP, Ponziani G. Natural history of cognitive dysfunction in multiple sclerosis: a 4-year longitudinal study. *Ital. J. Neurol. Sci.* 1998;19(6):S388-S391.

**4.** Amato MP, Ponziani G, Pracucci G, Bracco L, Siracusa G, Amaducci L. Cognitive impairment in early-onset multiple sclerosis. Pattern, predictors, and impact on everyday life in a 4-year follow-up. *Archives of Neurology.* 1995;52(2):168-172.

**5.** Amato MP, Ponziani G, Siracusa G, Sorbi S. Cognitive dysfunction in early-onset multiple sclerosis: a reappraisal after 10 years. *Archives of Neurology.* 2001;58(10):1602-1606.

**6.** Beckerman H, Kempen JC, Knol DL, Polman CH, Lankhorst GJ, de Groot V. The first 10 years with multiple sclerosis: the longitudinal course of daily functioning. *Journal of Rehabilitation Medicine.* 2013;45(1):68-75.

**7.** Borghi M, Carletto S, Ostacoli L, et al. Decline of Neuropsychological Abilities in a Large Sample of Patients with Multiple Sclerosis: A Two-Year Longitudinal Study. *Front. Hum. Neurosci.* 2016;10:11.

**8.** Bosma LV, Kragt JJ, Brieva L, et al. Progression on the Multiple Sclerosis Functional Composite in multiple sclerosis: what is the optimal cut-off for the three components? *Multiple Sclerosis.* 2010;16(7):862-867.

**9.** Bsteh G, Hegen H, Teuchner B, et al. Peripapillary retinal nerve fibre layer as measured by optical coherence tomography is a prognostic biomarker not only for physical but also for cognitive disability progression in multiple sclerosis. *Multiple Sclerosis.* 2019;25(2):196-203.

**10.** Chruzander C, Ytterberg C, Gottberg K, Einarsson U, Holmqvist LW, Johansson S. A 10-year follow-up of a population-based study of people with multiple sclerosis in Stockholm, Sweden: Changes in health-related quality of life and the value of different factors in predicting health-related quality of life. *J. Neurol. Sci.* 2014;339(1-2):57-63.

**11.** Crielaard L, Kavaliunas A, Ramanujam R, et al. Factors associated with and long-term outcome of benign multiple sclerosis: a nationwide cohort study. *Journal of Neurology, Neurosurgery & Psychiatry.* 2019;90(7):761-767.

**12.** de Groot V, Beckerman H, Uitdehaag BM, et al. Physical and cognitive functioning after 3 years can be predicted using information from the diagnostic process in recently diagnosed multiple sclerosis. *Arch Phys Med Rehabil.* 2009;90(9):1478-1488.

**13.** De Meijer L, Merlo D, Skibina O, et al. Monitoring cognitive change in multiple sclerosis using a computerized cognitive battery. *Multiple Sclerosis Journal - Experimental, Translational and Clinical.* 2018;4(4).

**14.** Demakis GJ, Buchanan RJ. Rated cognition in nursing home residents with multiple sclerosis: cross-sectional and longitudinal analyses. *Disability & Rehabilitation.* 2010;32(17):1438-1446.

**15.** Demakis GJ, Buchanan R, Dewald L. A longitudinal study of cognition in nursing home residents with multiple sclerosis. *Disability & Rehabilitation.* 2009;31(21):1734-1741.

**16.** Eijlers AJC, van Geest Q, Dekker I, et al. Predicting cognitive decline in multiple sclerosis: a 5-year follow-up study. *Brain.* 2018;141(9):2605-2618.

**17.** Fuchs TA, Wojcik C, Wilding GE, et al. Trait Conscientiousness predicts rate of longitudinal SDMT decline in multiple sclerosis. *Multiple Sclerosis.* 2020;26(2):245-252.

**18.** Healy BC, Barker L, Bakshi R, et al. Trajectories of Symbol Digit Modalities Test performance in individuals with multiple sclerosis. *Mult. Scler. J.* 2021;27(4):593-602.

**19.** Heled E, Aloni R, Achiron A. Cognitive functions and disability progression in relapsing-remitting multiple sclerosis: A longitudinal study. *Appl. Neuropsychol.-Adult.* 2021;28(2):210-219.

**20.** Hoogervorst EL, Kalkers NF, Uitdehaag BM, Polman CH. A study validating changes in the multiple sclerosis functional composite. *Archives of Neurology.* 2002;59(1):113-116.

**21.** Hughes AJ, Beier M, Hartoonian N, Turner AP, Amtmann D, Ehde DM. Self-efficacy as a longitudinal predictor of perceived cognitive impairment in individuals with multiple sclerosis. *Archives of Physical Medicine & Rehabilitation.* 2015;96(5):913-919.

**22.** Hughes AJ, Turner AP, Alschuler KN, et al. Association Between Sleep Problems and Perceived Cognitive Dysfunction Over 12 Months in Individuals with Multiple Sclerosis. *Behavioral Sleep Medicine.* 2018;16(1):79-91.

**23.** Jakimovski D, Zivadinov R, Ramanthan M, et al. Serum neurofilament light chain level associations with clinical and cognitive performance in multiple sclerosis: A longitudinal retrospective 5-year study. *Mult. Scler. J.* 2020;26(13):1670-1681.

**24.** Johansson S, Ytterberg C, Gottberg K, Holmqvist LW, von Koch L, Conradsson D. Participation in social/lifestyle activities in people with multiple sclerosis: Changes across 10 years and predictors of sustained participation. *Mult. Scler. J.* 2020;26(13):1775-1784.

**25.** Johnen A, Burkner PC, Landmeyer NC, et al. Can we predict cognitive decline after initial diagnosis of multiple sclerosis? Results from the German National early MS cohort (KKNMS). *Journal of Neurology.* 2019;266(2):386-397.

**26.** Jonsson A, Andresen J, Storr L, Tscherning T, Soelberg Sorensen P, Ravnborg M. Cognitive impairment in newly diagnosed multiple sclerosis patients: a 4-year follow-up study. *Journal of the Neurological Sciences.* 2006;245(1-2):77-85.

**27.** Lopez-Gongora M, Querol L, Escartin A. A one-year follow-up study of the Symbol Digit Modalities Test (SDMT) and the Paced Auditory Serial Addition Test (PASAT) in relapsing-remitting multiple sclerosis: an appraisal of comparative longitudinal sensitivity. *BMC Neurology.* 2015;15:40.

**28.** McKay KA, Manouchehrinia A, Berrigan L, Fisk JD, Olsson T, Hillert J. Long-term Cognitive Outcomes in Patients With Pediatric-Onset vs Adult-Onset Multiple Sclerosis. *JAMA Neurol.* 2019;76(9):1028-1034.

**29.** Motyl J, Friedova L, Vaneckova M, et al. Isolated Cognitive Decline in Neurologically Stable Patients with Multiple Sclerosis. *Diagnostics.* 2021;11(3):13.

**30.** Raimo S, Spitaleri D, Trojano L, Santangelo G. Apathy as a herald of cognitive changes in multiple sclerosis: A 2-year follow-up study. *Multiple Sclerosis.* 2020;26(3):363-371.

**31.** Roy S, Drake A, Fuchs T, et al. Longitudinal personality change associated with cognitive decline in multiple sclerosis. *Mult. Scler. J.* 2018;24(14):1909-1912.

**32.** Strober L, DeLuca J, Benedict RH, et al. Symbol Digit Modalities Test: A valid clinical trial endpoint for measuring cognition in multiple sclerosis. *Mult Scler.* 2019;25(13):1781-1790.

**33.** Uher T, Vaneckova M, Sormani MP, et al. Identification of multiple sclerosis patients at highest risk of cognitive impairment using an integrated brain magnetic resonance imaging assessment approach. *European Journal of Neurology.* 2017;24(2):292-301.

**34.** Wallach AI, Waltz M, Casper TC, et al. Cognitive processing speed in pediatric-onset multiple sclerosis: Baseline characteristics of impairment and prediction of decline. *Mult. Scler. J.* 2020;26(14):1938-1947.

**35.** Wu J, Jelinek GA, Weiland T, et al. Perceived cognitive impairment is associated with sexual dysfunction in people with multiple sclerosis: A 2.5-year follow-up study of a large international cohort. *Multiple Sclerosis and Related Disorders.* 2020;45:102410.

**36.** Ytterberg C, Johansson S, Andersson M, Holmqvist LW, von Koch L. Variations in functioning and disability in multiple sclerosis - A two-year prospective study. *J. Neurol.* 2008;255(7):967-973.

**37.** Benesova Y, Tvaroh A. Cognition and fatigue in patients with relapsing multiple sclerosis treated by subcutaneous interferon beta-1a: an observational study SKORE. *Ther. Adv. Neurol. Disord.* 2017;10(1):18-32.

**38.** Iaffaldano P, Viterbo RG, Paolicelli D, et al. Impact of natalizumab on cognitive performances and fatigue in relapsing multiple sclerosis: a prospective, open-label, two years observational study. *PLoS ONE [Electronic Resource].* 2012;7(4):e35843.

**39.** Koch MW, Mostert J, Repovic P, Bowen JD, Uitdehaag B, Cutter G. Is the Symbol Digit Modalities Test a useful outcome in secondary progressive multiple sclerosis? *Eur. J. Neurol.* 2021;28(6):2115-2120.

**40.** Patti F, Morra VB, Amato MP, et al. Subcutaneous interferon β-1a may protect against cognitive impairment in patients with relapsing-remitting multiple sclerosis: 5-year follow-up of the COGIMUS study. *PLoS One.* 2013;8(8):e74111.

**41.** Perumal J, Fox RJ, Balabanov R, et al. Outcomes of natalizumab treatment within 3 years of relapsing-remitting multiple sclerosis diagnosis: a prespecified 2-year interim analysis of STRIVE. *BMC Neurology.* 2019;19(1):116.

**42.** Rudick RA, Polman CH, Cohen JA, et al. Assessing disability progression with the Multiple Sclerosis Functional Composite. *Multiple Sclerosis.* 2009;15(8):984-997.

**43.** Schwartz CE, Kozora E, Zeng Q. Towards patient collaboration in cognitive assessment: Specificity, sensitivity, and incremental validity of self-report. *Ann. Behav. Med.* 1996;18(3):177-184.

**44.** Stephenson JJ, Kern DM, Agarwal SS, et al. Impact of natalizumab on patient-reported outcomes in multiple sclerosis: a longitudinal study. *Health & Quality of Life Outcomes.* 2012;10:155.

**45.** Benedict RHB, Tomic D, Cree BA, et al. Siponimod and Cognition in Secondary Progressive Multiple Sclerosis: EXPAND Secondary Analyses. *Neurology.* 2021;96(3):e376-e386.

**46.** Benedict RH, Cohan S, Lynch SG, et al. Improved cognitive outcomes in patients with relapsing-remitting multiple sclerosis treated with daclizumab beta: Results from the DECIDE study. *Multiple Sclerosis.* 2018;24(6):795-804.

**47.** Chan D, Binks S, Nicholas JM, et al. Effect of high-dose simvastatin on cognitive, neuropsychiatric, and health-related quality-of-life measures in secondary progressive multiple sclerosis: secondary analyses from the MS-STAT randomised, placebo-controlled trial. *Lancet Neurology.* 2017;16(8):591-600.

**48.** Cinar BP, Kosehasanogullari G, Yigit P, Ozakbas S. Cognitive dysfunction in patients with multiple sclerosis treated with first-line disease-modifying therapy: a multi-center, controlled study using the BICAMS battery. *Neurological Sciences.* 2017;38(2):337-342.

**49.** Comi G, Patti F, Rocca MA, et al. Efficacy of fingolimod and interferon beta-1b on cognitive, MRI, and clinical outcomes in relapsing-remitting multiple sclerosis: an 18-month, open-label, rater-blinded, randomised, multicentre study (the GOLDEN study). *J Neurol.* 2017;264(12):2436-2449.

**50.** DeLuca J, Schippling S, Montalban X, et al. Effect of Ozanimod on Symbol Digit Modalities Test Performance in Relapsing MS. *Multiple Sclerosis and Related Disorders.* 2021;48:102673.

**51.** De Giglio L, Marinelli F, Barletta VT, et al. Effect on Cognition of Estroprogestins Combined with Interferon Beta in Multiple Sclerosis: Analysis of Secondary Outcomes from a Randomised Controlled Trial. *CNS Drugs.* 2016;31(2):161-168.

**52.** Fischer JS, Priore RL, Jacobs LD, et al. Neuropsychological effects of interferon beta-1a in relapsing multiple sclerosis. *Ann. Neurol.* 2000;48(6):885-892.

**53.** Lincoln NB, Bradshaw LE, Constantinescu CS, et al. Group cognitive rehabilitation to reduce the psychological impact of multiple sclerosis on quality of life: the CRAMMS RCT. *Health Technology Assessment (Winchester, England).* 2020;24(4):1-182.

**54.** Patti F, Amato MP, Bastianello S, et al. Effects of immunomodulatory treatment with subcutaneous interferon beta-1a on cognitive decline in mildly disabled patients with relapsing-remitting multiple sclerosis. *Mult Scler.* 2010;16(1):68-77.

**55.** Schwid SR, Goodman AD, Weinstein A, McDermott MP, Johnson KP. Cognitive function in relapsing multiple sclerosis: minimal changes in a 10-year clinical trial. *J Neurol Sci.* 2007;255(1-2):57-63.

**56.** Weinstein A, Schwid SIL, Schiffer RB, McDermott MP, Giang DW, Goodman AD. Neuropsychologic status in multiple sclerosis after treatment with glatiramer. *Arch. Neurol.* 1999;56(3):319-324.

**57.** Weinstock-Guttman B, Galetta SL, Giovannoni G, et al. Additional efficacy endpoints from pivotal natalizumab trials in relapsing-remitting MS. *Journal of Neurology.* 2012;259(5):898-905.
